# Short-term treatment with high dose liraglutide improves lipid and lipoprotein profile and changes hormonal mediators of lipid metabolism in obese patients with no overt type 2 diabetes mellitus: a randomized, placebo-controlled, cross-over, double-blind clinical trial

**DOI:** 10.1186/s12933-019-0945-7

**Published:** 2019-10-31

**Authors:** Natia Peradze, Olivia M. Farr, Nikolaos Perakakis, Iolanda Lázaro, Aleix Sala-Vila, Christos S. Mantzoros

**Affiliations:** 1 Division of Endocrinology, Beth Israel Deaconess Medical Center/Harvard Medical School, 330 Brookline Ave, SL419, Boston, MA 02215 USA; 2 CIBER de Fisiopatología de la Obesidad y la Nutrición (CIBEROBN), Instituto de Salud Carlos III, Madrid, Spain; 3 Institut d’Investigacions Biomediques August Pi i Sunyer (IDIBAPS), Hospital Clinic of Barcelona, Villarroel 170, 08036 Barcelona, Spain; 4 Section of Endocrinology, Boston VA Healthcare System, Harvard Medical School, 150 South Huntington Avenue, Boston, MA 02130 USA

**Keywords:** Diabetes, Obesity, Liraglutide, GLP-1, Dyslipidemia, Lipoproteins, Metabolites, Metabolomics, Cardio-vascular disease

## Abstract

**Objective:**

Long-term treatment with up to 1.8 mg liraglutide improves cardiovascular and all-cause mortality in patients with type 2 diabetes at high risk for cardiovascular disease (CVD) and is currently under investigation in subjects without diabetes. Aim of our study was to investigate whether high dose (3 mg) short-term (5 weeks) treatment with liraglutide in obese patients with no overt type 2 diabetes affects metabolites, lipid and lipoprotein profile and components of activin–follistatin axis in cardiovascular beneficial or detrimental way.

**Research design and methods:**

Twenty obese patients participated in a randomized, placebo-controlled, cross-over, double-blind study and were administrated liraglutide 3 mg or placebo for 5 weeks. Metabolites, fatty acids, lipid–lipoprotein profile and concentrations of activins and follistatins (250 parameters) were assessed in serum at start and completion of each treatment.

**Results:**

Concentrations of important cardiovascular markers such as total, free and remnant cholesterol were reduced with liraglutide before and after adjusting for weight loss. Similarly, reductions in number of small and medium size LDL particles and in their total lipid concentration were observed with liraglutide and partially weight-loss related. Tyrosine levels were reduced and behenic acid levels were increased whereas only minor changes were observed in HDL, VLDL and IDL. Concentrations of activin AB and follistatin were significantly reduced in liraglutide-treated group.

**Conclusions:**

Treatment of obese patients without overt type 2 diabetes with high dose of liraglutide for a short period of time induces changes in lipid–lipoprotein and hormonal profile that are suggestive of lower risk of atherosclerosis and CVD.

*Trial registration* ClinicalTrials.gov Identifier: NCT02944500. Study ID Number 2015P000327. Registered November 2016

## Background

Liraglutide is a long-acting glucagon-like peptide (GLP) 1 receptor agonist (GLP-1RA) originally approved for treatment of type 2 diabetes mellitus at doses up to 1.8 mg and later for treatment of obesity at higher doses up to 3 mg daily [1, 2]. In general, liraglutide is known to be well-tolerated and the most common side effect is transient nausea [3, 4]. Liraglutide (at 1.8 mg) reduces the frequency of cardiovascular events and improves all-cause and cardiovascular mortality in patients with type 2 diabetes at high risk for cardiovascular disease (CVD) [5] and is currently under investigation among patients without diabetes. Several mechanisms have been proposed for the cardio-protective effects of liraglutide including reduction in inflammation and oxidative stress, reduction in microvascular thrombosis and improvement of endothelial function [6]. These effects may be partially resulting from the improved glycaemia and moderate weight loss observed after treatment with liraglutide but cannot fully explain the robust cardiovascular benefit [7, 8].

It has been suggested that improvement in lipid/lipoprotein metabolism which results in lower atherosclerosis may contribute to the cardio-protective effects of liraglutide. Dyslipidemia is characterized by high levels of circulating LDL and triglycerides and low HDL and is an established causally-related factor for the development of CVD [9]. Additionally, LDL, HDL, IDL and VLDL particle size and content as well as circulating levels of apolipoprotein B (apo B), remnant cholesterol and fatty acids have been associated with increased risk of CVD [10–13]. Recent studies in subjects treated with low dose (1.2 mg) liraglutide for 1-year for weight maintenance after a very low-calorie diet [14] or in subjects with type 2 diabetes treated with 1.8 mg liraglutide for 16 weeks and dietary counselling to achieve similar weight loss with the placebo group, reported changes in lipid profile indicative of a reduction in CVD risk [7]. However, it is unknown how early the changes in lipid profile occur with liraglutide treatment, whether they are dose-dependent and whether they are observed in patients with no overt type 2 diabetes and thus lower risk for CVD.

Additionally, it is unknown whether hormones serve as mediators of the lipid/lipoprotein changes observed with liraglutide treatment. Activins (activin A, B and AB) and follistatins (follistatin, follistatin-like 3 [FSTL3]) have recently emerged as important regulators of energy homeostasis, glucose and lipid metabolism [15–18]. Knockout of follistatin in hyperglycemic mice improves insulin sensitivity and reduces hepatic glucose output [18]. In humans, blood concentrations of follistatin and FSTL3 are positively associated with body mass index (BMI) and body fat percentage, whereas concentrations of follistatin are additionally positively associated with serum cholesterol and LDL-C [15]. Finally, early reduction of circulating follistatin after bariatric surgery predicts the improvement in insulin sensitivity observed later in morbidly obese individuals with and without type 2 diabetes. Follistatins are also the natural antagonists of activins. Activins are inhibitors of muscle growth [19, 20] and stimulators of obesity-related mitochondrial dysfunction [21] and their receptor has been targeted therapeutically, aiming to improve muscle mass and subsequently insulin sensitivity.

Aim of our study was to perform the first comprehensive untargeted metabolite analysis, with particular focus on lipid and lipoprotein profile, and to also evaluate possible hormonal mediators (activins and follistatins) in obese individuals with no overt type 2 diabetes treated with high doses of liraglutide (3 mg) or placebo for a very short period of time (5 weeks) in a cross-over design and assess: (a) whether metabolite or hormonal changes are observed after high dose short-term liraglutide treatment, (b) whether these changes are suggestive of a reduction in CVD risk, (c) whether these changes are independent of weight loss.

## Research design and methods

### Study characteristics

A randomized, placebo-controlled, cross-over, double-blind study was conducted in order to examine whether liraglutide 3 mg influences mechanisms underlying obesity and its comorbidities and identify target metabolites [3]. The study was performed in Beth Israel Deaconess Medical Center (BIDMC) MA, USA, with prior approval of Institutional Review Board (IRB) from 2016 till 2018. Clinical Trial Registration number is NCT02944500. Study was registered in November 2016. The study followed the Consolidated Standards of Reporting (CONSORT) guidelines and the International Conference on Harmonization for Good Clinical Practice [22] (Additional file 1: Consort Flow Diagram).

Potential participants for the study were recruited with advertisement and fliers and first screening was performed over the phone prior to the screening visit at BIDMC Clinical Research Center (CRC). IRB reviewed and approved advertisements prior to use, as well as the informed consent forms.

Inclusion criteria included BMI > 30 kg/m^2^ or BMI > 27 kg/m^2^ with comorbidities (such as insulin resistance, hypertension, dyslipidemia, cardiovascular disease, stroke and/or others). Exclusion criteria included breastfeeding, pregnancy, using metal intra-uterine device (IUD), changes in the dosage of hormonal contraceptive medications, impaired renal (creatinine clearance below 59 ml/min), heart or liver function, severe hypertriglyceridemia (triglycerides > 500 mg/dl), anemia (with haemoglobinless than 10 g/dl), gastroparesis or gallstones, inflammatory diseases, any uncontrolled endocrine condition or infectious diseases, taking any oral anti-diabetes agent except metformin, or the following medications: warfarin, steroids (inhaled or systemic due to reduced hypoglycemic effect), and subjects on other hormones (LHRH analogs etc.), hypersensitivity to the study medication, alcohol consumption (more than 210 g/week for men and more than 140 g/week for women), eating disorders, weight loss surgery or gastrectomy, personal or family history of multiple endocrine neoplasia (MEN) II or any cancer or lymphoma, history of diabetic ketoacidosis, subjects who cannot adhere to the experimental protocol for any reason.

At the screening visit interviews were conducted and personal information was collected according to the standardized ethics rules. Potential participants were explained the purpose and the procedures of the study, as well as risks and benefits. After subjects questions were answered, written informed consent was obtained for participation in this randomized, placebo-controlled, cross-over, double-blind study.

28 participants were enrolled in the study and randomized for phase 1 (Visits 1–6) to begin either liraglutide or placebo subcutaneously which was identical in appearance to liraglutide. Twenty subjects completed the study. Although type 2 diabetes was not an exclusion criterion, only three subjects were on treatment with metformin and had HbA1c below 6.5% (48 mmol/mol). All other subjects had no history of type 2 diabetes, had HbA1c < 6.5% (48 mmol/mol) and a fasting glucose < 126 mg/dl. In order to avoid and/or minimize side-effects, dose of liraglutide was titrated (from 0.6, 1.2, 1.8, 2.4 to 3.0 mg) for 5 weeks. Participants visited CRC weekly and examinations and blood draws were performed. Physician examined study subjects weekly and registered dietician reviewed food logs during the study course. Subjects were advised exercise (30 min 5 days a week) in combination with mild hypocaloric (reduced by 500 kcal) diet. Food/drink consumption, blood sugar levels and hunger levels on a visual analog scale were recorded. Dose of medication was self-administered at night and subjects continued to take their study medication at all times except from the washout period of approximately 3 weeks in between Phase 1 and Phase 2. After a wash-out period of approximately 3 weeks subjects returned to the research center for Phase 2 (visits 7–12) to repeat the same protocol with the opposite treatment to what they had received in Phase 1. Blood draws were performed by venipuncture by a registered nurse during visits at 1, 6, 7, 12 weeks (at the beginning and after each phase). Subjects were admitted at CRC the day before for overnight stay and venipuncture was performed at 8:30 a.m. After collection, samples were centrifuged and serum was stored at − 80 °C until NMR and gas-chromatography analysis.

### NMR-based metabolomics

Metabolic biomarkers were quantified from serum samples using high-throughput proton NMR metabolomics (Nightingale Health Ltd, Helsinki, Finland). This method provides simultaneous quantification of routine lipids, lipoprotein subclass profiling with lipid concentrations within 14 subclasses, fatty acid composition, and various low-molecular metabolites including amino acids, ketone bodies and gluconeogenesis-related metabolites in molar concentration units. Details of the experimentation and applications of the NMR metabolomics platform have been described previously [23].

### Quantification of serum fatty acids

Nonadecanoic acid (Merck) was introduced as internal standard in serum samples and fatty acids were converted to their corresponding fatty acid methyl esters (as described in [24] and separated by gas-chromatography using an Agilent HP 7890 Gas Chromatograph equipped with a 30 m × 0.25 μm × 0.25 mm SupraWAX-280 capillary column (Teknokroma, Barcelona, Spain), an autosampler, and a flame ionization detector.

### Biochemical measurements

Activin A, B and Activin AB, follistatin and FSTL3 were measured by enzyme-linked immunoassay (ELISA) (Ansh Laboratories, Webster, Texas) according to the manufacturer’s protocol.

### Statistical analysis

With 2 groups of 10 participants each, we estimated that our study would have 88% power for detecting difference in changes of molecules of interest and we would be able to detect an effect size difference of 0.9 at the α = 0.05 level. Harvard Catalyst biostatisticians produced randomization tables with SAS and delivered directly to the Research Pharmacy. This ensured blinding of study stuff, pharmacist and participants.

Data were analyzed using SPSS v25.0 (SPSS, Inc., Chicago, IL) for Windows, GraphPad prism 7 (GraphPad Software Inc., La Jolla, CA) and MetaboanalystR (https://www.metaboanalyst.ca) [25]. Data are reported as mean  ±  Standard Error of the Mean (SEM). Normality of distribution of the variables was assessed with Shapi–o-Wilks test. Outliers were identified with the use of ROUT method and a Q = 1% by Graphpad Prism 7 and removed from the analysis. On-treatment analysis was performed for all variables. Measurements were assessed in following ways: (a) Paired *t* test unadjusted and followed by analysis of covariance (ANCOVA) for weight loss for the delta changes between treatments (i.e. Delta Placebo = [Value at the end of placebo treatment − Baseline] vs. Delta Liraglutide = [Value at the end of liraglutide treatment − Baseline], (b) A sparse partial least squares discriminant analysis (sPLS-DA) was performed with the absolute values as well as with the delta changes to identify the factors that can best discriminate liraglutide vs. placebo group. For this analysis, missing values were replaced by a small value (half of the minimum positive value in the original dataset) and all data were mean-centered and divided by the standard deviation of each variable. The level of statistical significance was set at 0.05 for all analyses.

## Results

### Anthropometric characteristics

At baseline, there were no differences between the placebo and liraglutide groups in BMI (Placebo: 35.1 ± 5.6 kg/m^2^, Liraglutide: 35.5 ± 5.8 kg/m^2^, p = 0.08), triglycerides, VLDL, glucose and/or insulin levels in circulation. The placebo group had slightly higher cholesterol (data not presented).

No differences were detected between the groups in heart rate (bpm), systolic and diastolic blood pressures (mmHg) (*p* = 0.61, 0.37 and 0.44 respectively) [3].

### Side effects

Chi-square tests of the side effects revealed a significant difference between the placebo and liraglutide groups in self-reported decrease of appetite at 5 weeks (*p* = 0.03), while there were no differences found among the other side effects of the liraglutide compared with the placebo groups (Additional file 2: Table S1).

### Effects of treatment with liraglutide on cholesterols, triglycerides, lipid classes and apolipoproteins

Total, free and remnant cholesterol, as well as HDL3-C were lower with liraglutide treatment compared to placebo before and after adjusting for weight changes (Table 1). VLDL-C, LDL-C, HDL-C, HDL2-C and esterified cholesterol levels did not change with treatment. Similarly, no changes were observed in total triglycerides (TGs) or in TGs of VLDL, LDL or HDL. Among other lipid classes, only sphingomyelin levels were reduced with liraglutide, whereas no changes were observed in phosphoglycerides (PG), phosphatidylcholines (PC) and cholines. Regarding the apolipoprotein A1 (apoA1) and apoB that are associated with CVD risk, only apoB was slightly lower with liraglutide compared to placebo before and after adjusting for weight loss.

**Table 1:** Statistical analysis of delta changes of cholesterol and apolipoprotein subclasses in liraglutide- and placebo-treated individuals

Cholesterol	Placebo			Liraglutide			P^1^	P^2^
	Visit 1	Visit 6	Delta changes	Visit 1	Visit 6	Delta changes		
Total chol.	3.51 ± 0.78	3.69 ± 0.82	0.18 ± 0.09	3.44 ± 0.77	3.28 ± 0.73	− 0.17 ± 0.12	0.01	0.008
Free chol.	1.08 ± 0.24	1.13 ± 0.25	0.05 ± 0.02	1.06 ± 0.24	1.01 ± 0.23	− 0.05 ± 0.03	0.01	0.01
Remnant chol.	1.14 ± 0.26	1.24 ± 0.28	0.1 ± 0.04	1.11 ± 0.25	1.09 ± 0.24	− 0.03 ± 0.04	0.01	0.02
VLDL chol.	0.58 ± 0.13	0.64 ± 0.14	0.07 ± 0.02	0.55 ± 0.12	0.56 ± 0.13	0.01 ± 0.02	0.05	0.16
LDL chol.	1.35 ± 0.3	1.39 ± 0.31	− 0.04 ± 0.11	1.34 ± 0.3	1.24 ± 0.28	− 0.09 ± 0.06	0.62	0.57
HDL chol.	1.01 ± 0.22	1 ± 0.22	− 0.005 ± 0.02	0.99 ± 0.22	0.95 ± 0.21	− 0.05 ± 0.03	0.22	0.06
HDL2 chol.	0.55 ± 0.12	0.51 ± 0.11	− 0.06 ± 0.05	0.53 ± 0.12	0.5 ± 0.11	− 0.03 ± 0.02	0.61	0.91
HDL3-C (× 10^−2^)	46.2 ± 10.3	46.2 ± 10.3	0.02 ± 0.3	46.2 ± 10.3	44.9 ± 10	− 1.31 ± 0.39	0.01	0.01
Est. Chol.	2.43 ± 0.54	2.5 ± 0.56	− 0.06 ± 0.17	2.38 ± 0.53	2.27 ± 0.51	− 0.12 ± 0.09	0.73	0.72
Serum-TG	1.09 ± 0.24	1.07 ± 0.24	− 0.13 ± 0.11	1 ± 0.22	1.03 ± 0.23	0.02 ± 0.03	0.19	0.32
VLDL-TG	0.73 ± 0.16	0.71 ± 0.16	− 0.09 ± 0.08	0.66 ± 0.15	0.69 ± 0.16	0.03 ± 0.03	0.15	0.24
LDL-TG (× 10^−2^)	14.8 ± 3.3	14.9 ± 3.32	0.06 ± 0.44	14.1 ± 3.15	13.2 ± 2.96	− 0.87 ± 0.38	0.16	0.12
HDL-TG (× 10^−2^)	11.2 ± 2.5	11.9 ± 2.66	0.7 ± 0.41	10.7 ± 2.39	10.8 ± 2.41	0.12 ± 0.38	0.29	0.56
TotPG	1.42 ± 0.32	1.47 ± 0.33	0.05 ± 0.04	1.35 ± 0.3	1.34 ± 0.3	− 0.02 ± 0.04	0.22	0.21
PC	1.36 ± 0.3	1.42 ± 0.31	0.06 ± 0.03	1.3 ± 0.29	1.29 ± 0.29	− 0.01 ± 0.04	0.19	0.14
SM	0.43 ± 0.1	0.45 ± 0.1	0.01 ± 0.01	0.43 ± 0.1	0.41 ± 0.09	− 0.02 ± 0.01	0.03	0.01
TotCho	1.79 ± 0.4	1.84 ± 0.41	0.05 ± 0.04	1.73 ± 0.39	1.7 ± 0.38	− 0.03 ± 0.04	0.18	0.12
Apolipoproteins								
ApoA1 (g/L)	1.18 ± 0.26	1.2 ± 0.27	0.02 ± 0.01	1.17 ± 0.26	1.14 ± 0.26	− 0.03 ± 0.02	0.08	0.04
ApoB (g/L)	0.76 ± 0.17	0.81 ± 0.18	0.04 ± 0.02	0.74 ± 0.17	0.73 ± 0.16	− 0.02 ± 0.02	0.01	0.02
ApoB/ApoA1 (× 10^2^)	65.3 ± 14.6	67.6 ± 15.2	2.35 ± 1.2	64.4 ± 14.4	64.1 ± 14.3	− 0.29 ± 1.04	0.05	0.21

Values are given in mmol/L. P^1^—Delta statistics unadjusted. P^2^—Delta changes adjusted for weight change. Values are reported as mean ± SE. On treatment analysis was performed for all variables. The level of significance was set to p-value < 0.05 after Bonferroni correction. Baseline samples were collected at visit 1, post treatment samples were collected at visit 6

*Total chol* serum total cholesterol, *VLDL*-*C* total cholesterol in VLDL, *Remnant chol* remnant cholesterol (non-HDL, non-LDL -cholesterol), *LDL chol* total cholesterol in LDL, *HDL chol* total cholesterol in HDL, *HDL2*-*C* total cholesterol in HDL2, *HDL3*-*C* total cholesterol in HDL3, *EstC* Esterified cholesterol, *FreeC* free cholesterol, *Serum*-*TG* serum total triglycerides, *VLDL*-*TG* triglycerides in VLDL, *LDL*-*TG* triglycerides in LDL, *HDL*-*TG* triglycerides in HDL, *TotPG* total phosphoglycerides, *PC* phosphatidylcholine and other cholines, *SM* sphingomyelins, *TotCho* total cholines, *ApoA1* apolipoprotein A-I, *ApoB* apolipoprotein B, *ApoB/ApoA1* ratio of apolipoprotein B to apolipoprotein A-I

**Fig. 1:**
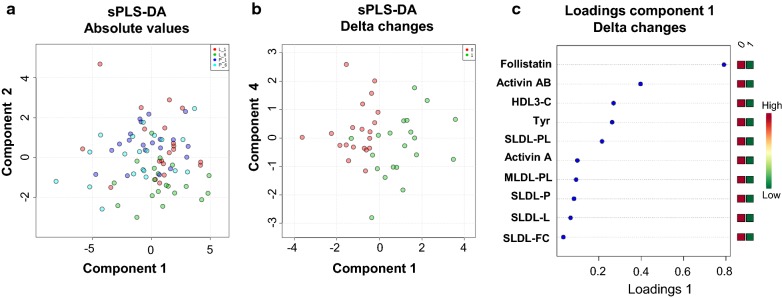
sPLS-DA comparing absolute and delta changes between liraglutide and placebo treatment. **a** Score plot of component 1 and component 2 of absolute values of 250 parameters (lipids, fatty acids, lipoproteins, aminoacids, activins and follistatins) before and after treatment with placebo or liraglutide. Dark blue dots depict the subjects before starting placebo treatment and red dots before starting liraglutide treatment. Light blue dots show the subjects 6 months after placebo treatment and green dots 6 months after liraglutide treatment. Component 1 and 2 consisting of 20 of the 250 parameters can partially identify when the subjects have received liraglutide (green dots gathered at low right) from the other three conditions (placebo before and after treatment, leptin before treatment, i.e. no separation between dark, light blue and red dots). **b** Score plot of component 1 and component 2 for delta changes (before–after) during placebo (red dots) and during liraglutide treatment (green dots). Component 1, consisting of 10 variables can discriminate well between the two conditions. **c** The ten parameters composing component 1 included hormones, lipoproteins and lipids

A sPLS-DA evaluating the absolute values and delta changes in all the measured metabolites (250 parameters) after liraglutide vs placebo treatment was performed (Fig. 1). The analysis showed that based on the observed changes in lipids-lipoproteins a partial discrimination between liraglutide vs placebo treatment can be achieved (Fig. 1a, b). HDL3-C was among the ten factors that its changes can best discriminate between liraglutide and placebo treatment (Fig. 1c).

### Effects of treatment with liraglutide on lipoprotein particles and content

The most robust changes with liraglutide in lipoprotein particles and content were observed in LDL (Table 2 for statistically significant parameters and Additional file 3: Table S2 for all the parameters). Concentrations of small and medium LDL particles were reduced with liraglutide compared to placebo, a finding that lost significance after adjusting for weight loss. Reductions in total lipids and phospholipids in small and medium LDL particles, as well as in free cholesterol in small LDL particles were also observed with liraglutide independently of weight loss. Despite these reductions in the absolute concentrations of the lipids with liraglutide, in the relative concentrations (as percent of the total lipid content in the particles) only modest changes were observed.

**Table 2:** Changes in lipoprotein subclasses after treatment with liraglutide vs. placebo

Lipoprotein subclasses	Placebo			Liraglutide			P^1^	P^2^
	Visit 1	Visit 6	Delta changes	Visit 1	Visit 6	Delta changes		
LDL								
Concentration of particles of different size								
S-LDL-P (× 10^−4^)	1.4 ± 0.31	1.47 ± 0.33	0.07 ± 0.04	1.37 ± 0.31	1.28 ± 0.29	− 0.09 ± 0.05	0.01	0.13
M-LDL-P (× 10^−4^)	1.2 ± 0.27	1.27 ± 0.28	0.07 ± 0.04	1.18 ± 0.26	1.11 ± 0.25	− 0.08 ± 0.05	0.02	0.15
Concentration of lipids in particles of different size								
S-LDL-L	0.39 ± 0.09	0.41 ± 0.09	0.02 ± 0.012	0.39 ± 0.09	0.36 ± 0.081	− 0.03 ± 0.01	0.01	0.01
S-LDL-PL (× 10^−2^)	12.5 ± 2.78	12.9 ± 2.87	0.42 ± 0.25	12.2 ± 2.72	11.6 ± 2.6	− 0.55 ± 0.25	0.01	0.01
S-LDL-FC (× 10^−2^)	7.23 ± 1.61	7.48 ± 1.67	0.25 ± 0.17	7.14 ± 1.6	6.75 ± 1.51	− 0.4 ± 0.2	0.02	0.01
M-LDL-L	0.61 ± 0.14	0.65 ± 0.14	0.03 ± 0.02	0.6 ± 0.14	0.57 ± 0.13	− 0.04 ± 0.02	0.02	0.01
M-LDL-PL	17.3 ± 3.86	18 ± 4.02	0.73 ± 0.4	16.9 ± 3.78	16.2 ± 3.62	− 0.75 ± 0.43	0.01	0.01
Concentration of lipids relative to total lipids in particles of different size								
S-LDL-PL_%	31.9 ± 7.12	31.4 ± 7.03	− 0.44 ± 0.44	31.9 ± 7.13	32.6 ± 7.28	0.65 ± 0.5	0.08	0.01
S-LDL-CE_%	43.2 ± 9.68	44 ± 9.85	0.81 ± 0.76	43.3 ± 9.68	42.4 ± 9.49	− 0.89 ± 0.8	0.12	0.02
S-LDL-FC_%	18.5 ± 4.12	18.2 ± 4.07	− 0.26 ± 0.14	18.6 ± 4.17	18.8 ± 4.21	0.19 ± 0.15	0.02	0.002
M-LDL-PL_%	28.4 ± 6.34	28.1 ± 6.28	− 0.29 ± 0.36	28.4 ± 6.35	29 ± 6.48	0.57 ± 0.43	0.11	0.02
M-LDL-CE_%	45 ± 10.1	46 ± 10.3	1 ± 0.7	45 ± 10.1	44.2 ± 9.89	− 0.81 ± 0.82	0.08	0.02
M-LDL-FC_%	19.8 ± 4.43	19.5 ± 4.36	− 0.34 ± 0.17	20 ± 4.48	20.3 ± 4.55	0.3 ± 0.24	0.02	0.002
HDL								
Concentration of lipids relative to total lipid concentration in the particles of different size								
L-HDL-C_%	44.9 ± 10	43.2 ± 9.63	− 1.52 ± 2.67	45.8 ± 10.2	45 ± 10.1	6.15 ± 3.55	0.03	0.12
L-HDL-CE_%	36.4 ± 8.11	35.2 ± 7.85	− 1.03 ± 2.31	36.6 ± 8.19	36.7 ± 8.21	5.59 ± 3.06	0.02	0.09
XL-HDL-PL_%	43.6 ± 9.82	43.5 ± 9.75	− 2.28 ± 1.88	44.5 ± 9.94	46.3 ± 10.4	1.76 ± 0.97	0.05	0.1
XL-HDL-CE_%	41.5 ± 9.2	42.4 ± 9.44	0.93 ± 0.91	40.6 ± 9.08	39.1 ± 8.75	− 1.43 ± 0.74	0.03	0.08
VLDL								
Concentration of particles of different size								
XS-VLDL-P (× 10^−4^)	0.35 ± 0.08	0.36 ± 0.08	0.016 ± 0.009	0.34 ± 0.08	0.33 ± 0.07	− 0.01 ± 0.01	0.04	0.31
Concentration of lipids in particles of different size								
XS-VLDL-L	0.44 ± 0.1	0.46 ± 0.1	0.02 ± 0.01	0.43 ± 0.1	0.42 ± 0.09	− 0.01 ± 0.01	0.04	0.04
XS-VLDL-PL (× 10^2^)	12.9 ± 2.88	13.5 ± 3.01	0.58 ± 0.39	12.6 ± 2.81	12.1 ± 2.71	− 0.42 ± 0.43	0.06	0.03
S-VLDL-C	17.3 ± 3.88	19.3 ± 4.32	1.98 ± 0.69	17 ± 3.8	17.2 ± 3.85	0.19 ± 0.78	0.04	0.08
S-VLDL-CE	0.11 ± 0.02	0.12 ± 0.03	0.01 ± 0.005	0.11 ± 0.02	0.11 ± 0.02	0.001 ± 0.01	0.04	0.07
IDL								
Concentrations of lipids relative to total lipid concentration in particles of different size								
IDL-P (× 10^−4^)	0.92 ± 0.2	0.93 ± 0.21	− 0.03 ± 0.06	0.9 ± 0.2	0.85 ± 0.19	− 0.05 ± 0.03	0.81	0.85
IDL-PL_%	27.8 ± 6.21	27.7 ± 6.18	− 0.12 ± 0.09	27.9 ± 6.23	27.9 ± 6.25	0.08 ± 0.11	0.11	0.15
IDL-C_%	61.6 ± 13.8	61.9 ± 13.8	3.37 ± 2.96	61.4 ± 13.7	61.1 ± 13.7	− 0.3 ± 0.43	0.25	0.01
IDL-CE_%	43.7 ± 9.78	44.4 ± 9.93	0.67 ± 0.22	43.7 ± 9.78	43.6 ± 9.75	− 0.12 ± 0.36	0.04	0.04

Values are given in mmol/L. P^1^—Delta statistics unadjusted. P^2^—Delta changes adjusted for changes in weight. Values are reported as mean ± SE. On treatment analysis was performed for all variables. The level of significance was set to p-value < 0.05 after Bonferroni correction. Baseline samples collected at visit 1, post treatment samples were collected at visit 6 (5 weeks of treatment total)

*S*-*LDL*-*P* concentration of small LDL particles, *M*-*LDL*-*P* concentration of medium LDL particles, *S*-*LDL*-*L* total lipids in small LDL, *S*-*LDL*-*PL* phospholipids in small LDL, *S*-*LDL*-*FC* free cholesterol in small LDL, *M*-*LDL*-*L* total lipids in medium LDL, *M*-*LDL*-*PL* phospholipids in medium LDL, *S*-*LDL*-*PL_%* phospholipids to total lipids ratio in small LDL, *S*-*LDL*-*CE_%* cholesterol esters to total lipids ratio in small LDL, *S*-*LDL*-*FC_%* free cholesterol to total lipids ratio in small LDL, *M*-*LDL*-*PL_%* phospholipids to total lipids ratio in medium LDL, *M*-*LDL*-*CE_%* cholesterol esters to total lipids ratio in medium LDL, *M*-*LDL*-*FC_%* free cholesterol to total lipids ratio in medium LDL, *L*-*HDL*-*C_%* total cholesterol to total lipids ratio in large HDL, *L*-*HDL*-*CE_%* cholesterol esters to total lipids ratio in large HDL, *XL*-*HDL*-*PL_%* phospholipids to total lipids ratio in very large HDL, *XL*-*HDL*-*CE_%* cholesterol esters to total lipids ratio in very large HDL, *XS*-*VLDL*-*PL* phospholipids to total lipids ratio in very small VLDL, *S*-*VLDL*-*C* total cholesterol in small VLDL, *S*-*VLDL*-*CE* cholesterol esters in small VLDL, *IDL*-*P* concentration of IDL particles, *IDL*-*PL* phospholipids in IDL, *IDL*-*C* total cholesterol in IDL, *IDL*-*CE* cholesterol esters in IDL

In terms of HDL, after liraglutide treatment and compared to placebo, only slightly higher relative concentrations of cholesterol and cholesterol esters in large HDL particles and lower of cholesterol esters in very large HDL were observed, but all the associations lost significance after adjusting for weight loss. Similarly only minor changes were observed in VLDL and IDL.

sPLS-DA indicated five parameters (all LDL-related) as good discriminating factors between liraglutide and placebo treatment (Fig. 1c). Specifically, these well-discriminating factors were the number of small size LDL-particles and their concentrations in lipids, free cholesterol and phospholipids and the concentration of phospholipids in medium size LDL-particles.

### Effects of treatment with liraglutide on fatty acids and aminoacids

The changes in fatty acids and aminoacids were generally mild (Table 3 and Additional file 3: Table S2). Before adjusting for weight loss, slightly lower concentrations of polyunsaturated fatty acids and specifically of omega-3 (i.e. Docosahexaenoic acid) and omega-6 fatty acids with liraglutide treatment were observed, but after adjusting only the reduction in omega-6 fatty acids remained significant. Similarly, among the 18 free fatty acids we investigated (Additional file 3: Table S2), only the concentrations of saturated behenic acid (C22:0) were higher with liraglutide compared to placebo before and after adjusting for weight loss (Table 3).

**Table 3:** Statistical analysis of delta changes of fatty acids, amino acids and activin/follistatins in liraglutide- and placebo-treated individuals

Fatty acids	Placebo			Liraglutide			P^1^	P^2^	P^3^	P^4^
	Visit 1	Visit 6	Delta changes	Visit 1	Visit 6	Delta changes				
TotFA	9.04 ± 2.01	9.47 ± 2.1	0.44 ± 0.25	8.65 ± 1.94	8.5 ± 1.9	− 0.16 ± 0.2	0.07	0.07		
UnSat (× 10^2^)	110 ± 24.6	112 ± 25	1.73 ± 0.84	110 ± 24.7	112 ± 25	1.22 ± 0.91	0.64	0.74		
DHA (× 10^2^)	8.7 ± 1.93	10 ± 2.21	1.75 ± 0.85	9.07 ± 2.03	8.77 ± 1.96	− 0.3 ± 0.46	0.04	0.09		
LA	2.5 ± 0.55	2.65 ± 0.59	0.15 ± 0.06	2.39 ± 0.54	2.38 ± 0.53	− 0.02 ± 0.08	0.07	0.1		
FAw3	0.28 ± 0.06	0.31 ± 0.07	0.04 ± 0.01	0.27 ± 0.06	0.27 ± 0.06	0.002 ± 0.02	0.04	0.11		
FAw6	3.02 ± 0.67	3.18 ± 0.71	0.16 ± 0.07	2.92 ± 0.65	2.87 ± 0.64	− 0.05 ± 0.08	0.03	0.04		
PUFA	3.3 ± 0.73	3.5 ± 0.78	0.2 ± 0.08	3.19 ± 0.71	3.14 ± 0.7	− 0.05 ± 0.1	0.03	0.05		
MUFA	2.27 ± 0.51	2.36 ± 0.52	0.09 ± 0.08	2.21 ± 0.49	2.17 ± 0.49	− 0.04 ± 0.06	0.23	0.15		
SFA	3.41 ± 0.76	3.53 ± 0.78	0.12 ± 0.1	3.25 ± 0.73	3.18 ± 0.71	− 0.07 ± 0.06	0.12	0.1		
C22:0	0.15 ± 0.01	0.14 ± 0.01	− 0.01 ± 0.01	0.14 ± 0.01	0.17 ± 0.01	0.02 ± 0.01	0.02	0.03		
Amino acids										
Tyrosine (× 10^2^)	6.15 ± 1.38	6.46 ± 1.44	0.31 ± 0.26	6.48 ± 1.45	5.77 ± 1.29	− 0.71 ± 0.25	0.03	0.001		
ELISAs										
Follistatin	3.43 ± 0.26	3.62 ± 0.25	0.01 ± 0.16	3.51 ± 0.32	2.7 ± 0.26	− 0.95 ± 0.24	0.007	0.004	0.02	0.81
FSTL3	12.5 ± 0.65	12.1 ± 0.48	− 1.03 ± 0.52	12.5 ± 0.68	12 ± 0.77	− 0.52 ± 0.6	0.52	0.31	0.67	0.88
Activin A	0.43 ± 0.02	0.37 ± 0.03	− 0.08 ± 0.02	0.43 ± 0.03	0.3 ± 0.02	− 0.12 ± 0.02	0.13	0.85	0.17	0.9
Activin B	96.6 ± 8.71	101 ± 8.5	4.51 ± 4.59	99.2 ± 10.2	109 ± 10.7	4.66 ± 3.27	0.98	0.95	0.72	0.65
Activin AB	3.26 ± 0.38	3.76 ± 0.41	0.52 ± 0.2	3.57 ± 0.46	2.94 ± 0.42	− 0.63 ± 0.29	0.04	0.004	0.03	0.1
Activin A/FST	0.14 ± 0.01	0.11 ± 0.01	− 0.03 ± 0.01	0.14 ± 0.01	0.13 ± 0.01	− 0.01 ± 0.01	0.26	0.03	0.33	0.52
Activin B/FST	31.6 ± 3.57	30.8 ± 3.33	− 1.01 ± 2.03	30.5 ± 2.79	41.8 ± 4.31	10.3 ± 2.45	0.01	0.03	0.02	0.09
ActivinAB/FST	1.21 ± 0.2	1.18 ± 0.15	− 0.03 ± 0.09	1.16 ± 0.17	1.24 ± 0.21	0.08 ± 0.13	0.43	0.54	0.46	0.13

Values are given in mmol/L. P^1^—Delta statistics unadjusted. P^2^—Delta changes adjusted for changes in weight. P^3^—Delta changes adjusted for changes in glucose. P^4^—Delta changes adjusted for the change in HOMA-IR index. Values are reported as mean ± SE. On treatment analysis was performed for all variables. The level of significance was set to p-value < 0.05 after Bonferroni correction. Baseline samples collected at visit 1, post treatment samples were collected at visit 6 (5 weeks of treatment total)

*TotFA* total fatty acids, *UnSat* unsaturated fatty acids, *DHA* 22:6, docosahexaenoic acid, *LA* 18:2, linoleic acid, *FAw3* omega-3 fatty acids, *FAw6* omega-6 fatty acids, *PUFA* polyunsaturated fatty acids, *MUFA* monounsaturated fatty acids, *SFA* saturated fatty acids, *C22:0* behenic acid

Among nine amino acids included in our analysis, tyrosine was the only amino acid the level of which was significantly lower before and after adjusting for weight loss in the liraglutide treatment. Tyrosine was also identified as important discriminating factors in the sPLS-DA between groups (Fig. 1).

### Effects of treatment with liraglutide on circulating levels of activins/follistatins

Liraglutide treatment did not affect circulating levels of Activin A, Activin B and FSTL3. In contrast, the concentrations of follistatin and activin AB were significantly lower in treated group versus placebo (~ 23% for follistatin and ~ 17% for Activin AB, Table 3). The significance was maintained after adjustment for weight loss or glucose but was lost after adjustment for insulin resistance (HOMA-IR) (Activin AB, p = 0.103 and follistatin p = 0.81, respectively). Follistatin, activin AB were also recognized in sPLS-DA as important factors for discriminating between placebo and liraglutide treatments.

Changes in follistatin correlated strongly with changes in glucose (r = 0.4, p = 0.039), while activin AB correlated strongly with HOMA index (r = 0.41, p = 0.009), but not with lipoproteins (data not shown).

## Discussion

We demonstrate herein that treatment with high dose liraglutide for a short period of time (5 weeks) in obese patients without overt type 2 diabetes initiates significant alterations in lipid and lipoprotein profile compared to placebo. These alterations are partially independent of weight loss, they are similar to changes observed after long-term treatment with liraglutide in patients with type 2 diabetes and at high risk for CVD and they are indicative of a potential cardiovascular benefit [26]. Additionally, we demonstrate that hormones (i.e. follistatin and activin AB) that have been recently related to lipid metabolism, insulin resistance and glucose regulation are significantly reduced after liraglutide treatment, supporting their role as hormonal mediators of metabolic procedures in humans.

First, we observe lower levels of total, free and remnant cholesterol and apoB with liraglutide compared to placebo [1]. All these molecules are considered established markers of CVD mainly by accelerating atherogenic processes [27]. Remnant cholesterol was also reduced postprandially in patients with type 2 diabetes treated with 1.8 mg liraglutide for 16 weeks versus placebo [7]. In agreement with our findings, a recent study demonstrated significant improvement in lipid and lipoprotein profiles as a result of 14 weeks treatment with liraglutide combined with modest restriction of caloric intake [28]. Reductions in apoB have also been reported in a study including obese subjects that had initially undergone a very low-calorie diet for 8 weeks followed by treatment with liraglutide in low dose (1.2 mg) for 1 year vs. meal replacements for weight maintenance [14]. Similarly, reduced apoB48 (isoform of apoB) levels were observed after a fat-rich meal in patients with type 2 diabetes treated with 1.8 mg liraglutide [29]. The changes we observe are generally more modest compared with others, which is probably explained by the shorter duration of treatment. The mechanism involved in these changes remains unknown, albeit it has been suggested, that liraglutide may lead to reduced secretion of chylomicrons containing apoB48 and thus to decreased absorption of triglycerides [29]. In contrast to LDL, the changes we observed in HDL, VLDL and IDL were minor and do not point to a clear direction regarding their associations to cardiovascular outcome, which might be due to the relatively short duration of the study, while significant reduction of total cholesterol, LDL cholesterol, TG and non-high-density cholesterol has been demonstrated in a 14-week liraglutide treatment and associated with improvements in CVD risks and outcomes [28]. It has been suggested that liraglutide treatment might be supporting a shift from lipids to glucose oxidation, resulting in lower glucose levels and reduction in lipolysis and lipid oxidation [30].

Regarding lipid subclasses, we observed changes primarily in LDL concentrations. Firstly, the numbers of small and medium particles (S-LDL-P, M-LDL-P) were lower after liraglutide treatment compared to placebo. Number of LDL particles (LDL-P) has been positively associated with increased CVD risk and in several large studies this association was stronger than LDL-C [31–33]. Additionally, a discordance between LDL-C levels and LDL-P or apolipoprotein B is often observed in the general population and in these cases, LDL-P and apolipoprotein B can be used to improve the assessment of CVD risk [33, 34]. Regarding particle size, small LDL particles are considered more atherogenic, since they have a longer circulation time and higher ability at penetrating the arterial wall [35]. Studies have also shown, that small LDL and cholesterol in small LDL are reliable markers of CVD [10, 36]. Thus, the lower levels of LDL-P and particularly of small LDL-P that we observe with liraglutide treatment point to a reduced CVD risk with liraglutide treatment, albeit these changes may be related to the concomitant weight loss. In a mechanistic study involving a 7-week treatment of db/db mice, liraglutide inhibited expression of proprotein convertase subtilisin/kexin type 9 (PCSK9) and low-density lipoprotein receptor (LDLR) in liver and accordingly the concentration of PCSK9 in serum and improved hepatic lipid accumulation. PCSK9 is known to play the key role in regulation of cholesterol homeostasis and is associated with glucose metabolism. Based on the mention study, PCSK9 might be one of the possible pathways through which liraglutide mediates its lipid-lowering effect [37].

Regarding the lipid content of the LDL particles, we observe a reduction in absolute concentrations of total lipids, phospholipids and free cholesterol in small LDL-P as well as in total lipids and phospholipids in medium LDL-P with liraglutide treatment and independently of weight loss. These reductions may be related to the lower number of small and medium LDL-P and consequently the lower amount of total lipids in LDL with liraglutide. When analyzed as relative concentrations to total lipids, we observe minor changes pointing towards higher free cholesterols and phospholipids and lower cholesterol esters in small and/or medium LDLs compared to placebo. Phospholipids together with free cholesterol and apolipoproteins are forming the outer hydrophilic layer of the lipoprotein which enables transportation of the hydrophobic cholesterol esters and triglycerides located in the inner part. Whether longer duration of liraglutide treatment affects the relative lipid content of LDLs and consequently their structure and metabolism have not been investigated yet.

Regarding lipid classes and fatty acids, sphingomyelins were lower with liraglutide treatment compared to placebo independently of weight loss. Interestingly, levels of two individual sphingomyelin species have been previously positively associated with CVD risk in a large prospective study (Bruneck study) [38]. Our study design did not allow us to identify which specific sphingomyelin species are reduced with liraglutide. In contrast, we did not observe any changes in phosphatidylcholines, phosphatidylglycerols or total cholines. Regarding fatty acids, slightly lower concentrations of PUFA, omega-3 and omega-6 fatty acids were observed, which were partially weight-loss related. An interesting effect of liraglutide treatment was the raise of behenic acid (C22:0), which lasted even after adjusting for weight loss. There is increasing epidemiologic evidence on the cardiometabolic benefits of increasing circulating concentrations of this very-long chain saturated fatty acid, the status of which is marginally influenced by its dietary intake. Specifically, higher levels of behenic acid are associated with a lower risk for diabetes, even after adjusting for diabetes risk factors and adiposity [39] as well as with lower risk of incident heart failure [40] and lower incidence of coronary heart disease (CHD) [41].

Studies of liraglutide and metformin combination have shown that liraglutide treatment might be more effective for reduction in non-estherified fatty acids (NEFA) and LDL profile in patients with newly diagnosed type 2 diabetes compared with metformin due to its ability to enhance insulin secretion and inhibit inflammatory cytokines [30, 42].

We also observe a reduction in circulating tyrosine levels with liraglutide. Tyrosine is a non-essential amino acid that is involved in signal transduction mechanisms. Phosphorylation of tyrosine through tyrosine kinases and dephosphorylation by protein tyrosine phosphatases are part of signaling pathways that are implicated in fundamental cellular functions, including cell growth, differentiation and oncogenic transformation [43]. Significant elevation of tyrosine has been identified in individuals with metabolic disorders [44] and had highly significant associations with future diabetes [45]. Tyrosine kinase inhibitors used for treatment of malignancies have demonstrated improvement of glycaemia in cases of type 1 diabetes and type 2 diabetes, possibly by enhancing beta cell survival and insulin secretion [46]. However, a clear link between circulating tyrosine levels and insulin resistance, diabetes or CVD risk has not been established yet.

Finally, we observe a significant reduction in follistatin and in activin AB. Follistatin is mainly secreted by the liver and its inactivation in hyperglycemic mice improves glucose homeostasis by reducing insulin resistance [18]. We have recently demonstrated that glucose or the concomitant insulin release during OGTT reduces follistatin levels, suggesting the presence of a feedback loop mechanism controlling the effects of follistatin on glucose metabolism [16]. In line with these findings, we have also reported a reduction of follistatin in morbidly obese patients early after bariatric surgery, which predicts the improvement in insulin sensitivity observed later in these patients. In this study, we expand our findings by showing that short term treatment with liraglutide reduces robustly circulating follistatin. This reduction is weight-independent but HOMA-IR dependent, confirming the link of follistatin with insulin sensitivity. Follistatin is also the natural inhibitor of activins. Although, activin A and B do not change with liraglutide treatment, a robust decrease of activin AB is observed. Activin AB is a heterodimer of inhibin βA and inhibin βB, which as dimers form activin A and activin B respectively [47]. Thus, the explicit reduction in activin AB and not in A or B indicates the involvement of mechanisms downstream of gene expression and probably in mechanisms defining heterodimer assembly and secretion. In contrast to insulin sensitivity, both the absolute as well as the delta changes of follistatin and activin AB were not associated with the changes observed in lipid–lipoprotein profile arguing against a significant involvement of these hormones in lipid metabolism. Future mechanistic studies should aim to investigate whether a direct GLP-1/Follistatin–Activin AB crosstalk exists that contributes to the regulation of glucose homeostasis.

The current study has some strengths and limitations. A major strength is the double-blinded placebo controlled cross-over design which reduces potential confounders and increases power. Additionally, the measurements were performed from personnel blinded for treatment groups. A limitation is the lack of postprandial blood draws, which would have been important mainly for parameters participating in the exogenous pathway of lipid metabolism (i.e. chylomicrons). Finally, our study was focused on lipoproteins and large lipid classes and did not include an untargeted analysis of individual lipid species.

## Conclusion

Our study shows that treatment of obese patients without overt type 2 diabetes with high dose of liraglutide for a short period of time induces changes in lipoprotein and lipid profile that are associated with lower risk of atherosclerosis and CVD and reduces the blood levels of activin AB and its antagonist, i.e. follistatin, a hormone recently identified as a potentially major player in insulin sensitivity.

## Supplementary information

**Additional file 1.** CONSORT 2010 Flow Diagram.

**Additional file 2: Table S1.** Chi-square tests of the side effects in placebo and liraglutide groups by week 5 of treatment. Information about self-reported adverse events in liraglutide- and placebo-treated individuals.

**Additional file 3: Table S2:** Statistical analysis of delta changes of metabolites in liraglutide- and placebo-treated individuals. Information about delta changes in all metabolites measured in liraglutide- and placebo-treated individuals.

## Data Availability

The dataset(s) supporting the conclusions of this article is(are) included within the article (and its additional file(s)).

## Abbreviations

CVD: cardiovascular disease
LDL: low-density lipoproteins
HDL: high-density lipoproteins
VLDL: very-low density lipoproteins
IDL: intermediate-density lipoproteins
apoB: apolipoprotein B
CONSORT: Consolidated Standards of Reporting
BIDMC: Beth Israel Deaconess Medical Center
IRB: Institutional Review Board
CRC: Clinical Research Center
BMI: body mass index
Hgb: hemoglobin
IUD: intra-uterine device
LHRH: luteinizing hormone-releasing hormone
MEN: multiple endocrine neoplasia
NMR: nuclear magnetic resonance
Total chol: serum total cholesterol
VLDL-C: total cholesterol in VLDL
Remnant chol: remnant cholesterol (non-HDL, non-LDL-cholesterol)
LDL chol: total cholesterol in LDL
HDL chol: total cholesterol in HDL
HDL2-C: total cholesterol in HDL2
HDL3-C: total cholesterol in HDL3
EstC: Esterified cholesterol
FreeC: free cholesterol
Serum-TG: serum total triglycerides
VLDL-TG: triglycerides in VLDL
LDL-TG: triglycerides in LDL
HDL-TG: triglycerides in HDL
TotPG: total phosphoglycerides
PC: phosphatidylcholine and other cholines
SM: sphingomyelins
TotCho: total cholines
ApoA1: apolipoprotein A-I
ApoB: apolipoprotein B
ApoB/ApoA1: ratio of apolipoprotein B to apolipoprotein A-I
S-LDL-P: concentration of small LDL particles
M-LDL-P: concentration of medium LDL particles
S-LDL-L: total lipids in small LDL
S-LDL-PL: phospholipids in small LDL
S-LDL-FC: free cholesterol in small LDL
M-LDL-L: total lipids in medium LDL
M-LDL-PL: phospholipids in medium LDL
S-LDL-PL_%: phospholipids to total lipids ratio in small LDL
S-LDL-CE_%: cholesterol esters to total lipids ratio in small LDL
S-LDL-FC_%: free cholesterol to total lipids ratio in small LDL
M-LDL-PL_%: phospholipids to total lipids ratio in medium LDL
M-LDL-CE_%: cholesterol esters to total lipids ratio in medium LDL
M-LDL-FC_%: free cholesterol to total lipids ratio in medium LDL
L-HDL-C_%: total cholesterol to total lipids ratio in large HDL
L-HDL-CE_%: cholesterol esters to total lipids ratio in large HDL
XL-HDL-PL_%: phospholipids to total lipids ratio in very large HDL
XL-HDL-CE_%: cholesterol esters to total lipids ratio in very large HDL
XS-VLDL-PL: phospholipids to total lipids ratio in very small VLDL
S-VLDL-C: total cholesterol in small VLDL
S-VLDL-CE: cholesterol esters in small VLDL
IDL-P: concentration of IDL particles
IDL-PL: phospholipids in IDL
IDL-C: total cholesterol in IDL
IDL-CE: cholesterol esters in IDL
TotFA: total fatty acids
UnSat: unsaturated fatty acids
DHA: 22:6n3, docosahexaenoic acid
LA: 18:2n6, linoleic acid
FAw3: omega-3 fatty acids
FAw6: omega-6 fatty acids
PUFA: polyunsaturated fatty acids
MUFA: monounsaturated fatty acids
SFA: saturated fatty acids
C22:0: behenic acid
NEFA: non-esterified fatty acids
PCSK9: proprotein convertase subtilisin/kexin type 9
LDLR: low-density lipoprotein receptor
