# Supplementary material for: Short-term treatment with high dose liraglutide improves lipid and lipoprotein profile and changes hormonal mediators of lipid metabolism in obese patients with no overt type 2 diabetes mellitus: a randomized, placebo-controlled, cross-over, double-blind clinical trial
Source: Cardiovasc Diabetol. 2019 Oct 31;18:141. doi: 10.1186/s12933-019-0945-7 (PMC6823961; doi:10.1186/s12933-019-0945-7)
Supplement: Supplementary file 1 — Additional file 1. CONSORT 2010 Flow Diagram. [file 12933_2019_945_MOESM1_ESM.docx]

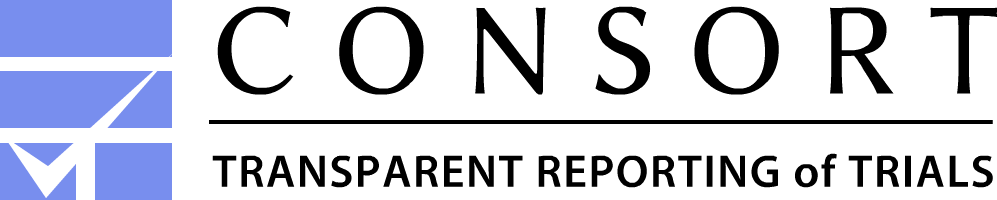


**CONSORT 2010 Flow Diagram**

Analysed (n=10)
♦ Excluded from analysis (n=0)

Analysed (n=10)
♦ Excluded from analysis (n=0)

Lost to follow-up (no reason given) (n=3)

Discontinued intervention (MSSI change) (n=1)

Lost to follow-up (no reason given) (n=2)

Discontinued intervention (was no longer interested, n=1; metal dental fragment was discovered, n=1)

## Follow-Up

## Analysis

## Enrollment

Allocated to Liraglutide first, Placebo second (n=14)

♦ Received allocated intervention (n=14)

♦ Did not receive allocated intervention (n=0)

## Allocation

Allocated to Placebo first, Liraglutide second (n=14)

♦ Received allocated intervention (n=14)

♦ Did not receive allocated intervention (n=4)

Randomized (n=28)

Excluded (n=15)

♦  Not meeting inclusion criteria (n=9)

♦  Declined to participate (n=1)

♦  Other reasons (n=5)

Assessed for eligibility (n=44)
