# Supplementary material for: Short-term treatment with high dose liraglutide improves lipid and lipoprotein profile and changes hormonal mediators of lipid metabolism in obese patients with no overt type 2 diabetes mellitus: a randomized, placebo-controlled, cross-over, double-blind clinical trial
Source: Cardiovasc Diabetol. 2019 Oct 31;18:141. doi: 10.1186/s12933-019-0945-7 (PMC6823961; doi:10.1186/s12933-019-0945-7)
Supplement: Supplementary file 2 — Additional file 2: Table S1. Chi-square tests of the side effects in placebo and liraglutide groups by week 5 of treatment. Information about self-reported adverse events in liraglutide- and placebo-treated individuals. [file 12933_2019_945_MOESM2_ESM.docx]

Table S1 – Adverse events

| **Event** | **Placebo (5 wks) (20 total)** | **Liraglutide (5 wks)**  **(20 total)** | **P value** |
| --- | --- | --- | --- |
| Headache | 0 | 3 | 0.07 |
| Dizziness | 0 | 2 | 0.15 |
| Tiredness | 0 | 2 | 0.15 |
| Nausea | 0 | 3 | 0.07 |
| Intolerable nausea | 0 | 0 |  |
| Vomiting | 0 | 0 |  |
| Low Blood Sugar | 0 | 0 |  |
| Dry Mouth | 0 | 0 |  |
| Constipation | 0 | 2 | 0.15 |
| Diarrhea | 1 | 0 | 0.30 |
| Abdominal Pain | 1 | 1 | 1.00 |
| Decreased Appetite | 2 | 8 | **0.03** |
| Upset Stomach | 0 | 2 | 0.15 |

Chi-square tests of the side effects in placebo and liraglutide groups by week 5 of treatment.
