# Supplementary material for: Short-term treatment with high dose liraglutide improves lipid and lipoprotein profile and changes hormonal mediators of lipid metabolism in obese patients with no overt type 2 diabetes mellitus: a randomized, placebo-controlled, cross-over, double-blind clinical trial
Source: Cardiovasc Diabetol. 2019 Oct 31;18:141. doi: 10.1186/s12933-019-0945-7 (PMC6823961; doi:10.1186/s12933-019-0945-7)
Supplement: Supplementary file 3 — Additional file 3: Table S2: Statistical analysis of delta changes of metabolites in liraglutide- and placebo-treated individuals. Information about delta changes in all metabolites measured in liraglutide- and placebo-treated individuals. [file 12933_2019_945_MOESM3_ESM.docx]

Table S2– Statistical analysis of delta changes of metabolites in liraglutide- and placebo- treated individuals.

|  | **Placebo** | | | | | |  |  |  | **Liraglutide** | | | | | |  |  |  | **P^1^** | **P^2^** |
| --- | --- | --- | --- | --- | --- | --- | --- | --- | --- | --- | --- | --- | --- | --- | --- | --- | --- | --- | --- | --- |
|  | **Visit 1** | | | **Visit 6** | | | **Delta changes** | | | **Visit 1** | | | **Visit 6** | | | **Delta changes** | | |  |  |
| **Lipoprotein subclasses** |  |  |  |  |  |  |  |  |  |  |  |  |  |  |  |  |  |  |  |  |
| XXL-VLDL-P (x10^^-10^) | 1.37 | ± | 0.10 | 1.52 | ± | 0.15 | 0.07 | ± | 0.12 | 1.25 | ± | 0.16 | 1.26 | ± | 0.10 | 0.01 | ± | 0.14 | 0.73 | 0.65 |
| XXL-VLDL-L (x10^^-2^) | 2.86 | ± | 0.22 | 3.20 | ± | 0.33 | 0.16 | ± | 0.26 | 2.77 | ± | 0.32 | 2.64 | ± | 0.21 | 0.02 | ± | 0.29 | 0.72 | 0.35 |
| XXL-VLDL-PL (x10^^-2^) | 0.29 | ± | 0.03 | 0.32 | ± | 0.04 | 0.02 | ± | 0.03 | 0.28 | ± | 0.04 | 0.25 | ± | 0.03 | -0.01 | ± | 0.04 | 0.54 | 0.52 |
| XXL-VLDL-C (x10^^-2^) | 0.41 | ± | 0.04 | 0.49 | ± | 0.06 | 0.05 | ± | 0.04 | 0.37 | ± | 0.05 | 0.40 | ± | 0.03 | 0.02 | ± | 0.05 | 0.65 | 0.54 |
| XXL-VLDL-CE (x10^^-2^) | 0.26 | ± | 0.03 | 0.34 | ± | 0.04 | 0.08 | ± | 0.03 | 0.25 | ± | 0.03 | 0.27 | ± | 0.02 | 0.02 | ± | 0.03 | 0.27 | 0.60 |
| XXL-VLDL-FC (x10^^-2^) | 0.14 | ± | 0.02 | 0.17 | ± | 0.02 | 0.01 | ± | 0.02 | 0.13 | ± | 0.02 | 0.12 | ± | 0.02 | 0.02 | ± | 0.02 | 0.64 | 0.52 |
| XXL-VLDL-TG (x10^^-2^) | 2.17 | ± | 0.15 | 2.39 | ± | 0.23 | 0.09 | ± | 0.19 | 1.98 | ± | 0.25 | 1.99 | ± | 0.15 | 0.01 | ± | 0.21 | 0.76 | 0.30 |
| XL-VLDL-P (x10^^-10^) | 5.13 | ± | 0.60 | 5.49 | ± | 0.53 | -0.49 | ± | 0.88 | 4.54 | ± | 0.73 | 4.72 | ± | 0.53 | 0.39 | ± | 0.58 | 0.27 | 0.62 |
| XL-VLDL-L (x10^^-2^) | 4.98 | ± | 0.59 | 5.37 | ± | 0.51 | -0.44 | ± | 0.85 | 4.19 | ± | 0.73 | 4.60 | ± | 0.51 | 0.37 | ± | 0.57 | 0.30 | 0.29 |
| XL-VLDL-PL (x10^^-2^) | 0.71 | ± | 0.10 | 0.75 | ± | 0.09 | -0.03 | ± | 0.14 | 0.61 | ± | 0.12 | 0.64 | ± | 0.09 | 0.03 | ± | 0.10 | 0.67 | 0.62 |
| XL-VLDL-C (x10^^-2^) | 1.09 | ± | 0.12 | 1.17 | ± | 0.12 | -0.04 | ± | 0.17 | 0.97 | ± | 0.15 | 1.07 | ± | 0.09 | 0.09 | ± | 0.13 | 0.40 | 0.36 |
| XL-VLDL-CE (x10^^-2^) | 0.71 | ± | 0.07 | 0.81 | ± | 0.09 | 0.06 | ± | 0.07 | 0.63 | ± | 0.09 | 0.72 | ± | 0.05 | 0.08 | ± | 0.07 | 0.83 | 0.24 |
| XL-VLDL-FC (x10^^-2^) | 0.39 | ± | 0.05 | 0.42 | ± | 0.05 | -0.01 | ± | 0.08 | 0.37 | ± | 0.07 | 0.35 | ± | 0.05 | 0.01 | ± | 0.06 | 0.85 | 0.73 |
| XL-VLDL-TG (x10^^-2^) | 3.18 | ± | 0.38 | 3.33 | ± | 0.33 | -0.36 | ± | 0.54 | 2.61 | ± | 0.46 | 2.89 | ± | 0.33 | 0.26 | ± | 0.35 | 0.21 | 0.21 |
| L-VLDL-P (x10^^-10^) | 36.7 | ± | 3.45 | 36.2 | ± | 3.19 | -4.07 | ± | 5.32 | 32.1 | ± | 3.59 | 34.0 | ± | 3.04 | 3.46 | ± | 2.68 | 0.16 | 0.54 |
| L-VLDL-L (x10^^-2^) | 21.0 | ± | 2.00 | 20.8 | ± | 1.85 | -2.29 | ± | 3.07 | 18.4 | ± | 2.08 | 19.5 | ± | 1.76 | 1.98 | ± | 1.55 | 0.16 | 0.17 |
| L-VLDL-PL (x10^^-2^) | 3.64 | ± | 0.36 | 3.62 | ± | 0.33 | -0.38 | ± | 0.55 | 3.18 | ± | 0.37 | 3.35 | ± | 0.32 | 0.31 | ± | 0.27 | 0.21 | 0.22 |
| L-VLDL-C (x10^^-2^) | 4.87 | ± | 0.46 | 4.96 | ± | 0.45 | -0.41 | ± | 0.71 | 4.34 | ± | 0.49 | 4.63 | ± | 0.40 | 0.50 | ± | 0.38 | 0.19 | 0.19 |
| L-VLDL-CE (x10^^-2^) | 3.14 | ± | 0.24 | 3.25 | ± | 0.25 | -0.23 | ± | 0.39 | 2.90 | ± | 0.25 | 3.12 | ± | 0.20 | 0.36 | ± | 0.21 | 0.13 | 0.12 |
| L-VLDL-FC (x10^^-2^) | 1.72 | ± | 0.23 | 1.71 | ± | 0.21 | -0.19 | ± | 0.32 | 1.44 | ± | 0.24 | 1.51 | ± | 0.20 | 0.14 | ± | 0.17 | 0.30 | 0.33 |
| L-VLDL-TG (x10^^-2^) | 12.5 | ± | 1.19 | 12.2 | ± | 1.09 | -1.50 | ± | 1.82 | 10.9 | ± | 1.24 | 11.5 | ± | 1.06 | 1.17 | ± | 0.92 | 0.15 | 0.14 |
| M-VLDL-P (x10^^-10^) | 142 | ± | 9.53 | 142 | ± | 9.40 | -14.6 | ± | 16.7 | 130 | ± | 9.21 | 138 | ± | 8.43 | 8.26 | ± | 5.95 | 0.18 | 0.42 |
| M-VLDL-L (x10^^-2^) | 47.0 | ± | 3.15 | 47.0 | ± | 3.11 | -4.71 | ± | 5.56 | 43.0 | ± | 3.06 | 45.7 | ± | 2.79 | 2.73 | ± | 1.99 | 0.19 | 0.31 |
| M-VLDL-PL (x10^^-2^) | 9.51 | ± | 0.61 | 9.53 | ± | 0.59 | -0.93 | ± | 1.10 | 8.76 | ± | 0.60 | 9.22 | ± | 0.54 | 0.46 | ± | 0.38 | 0.22 | 0.36 |
| M-VLDL-C (x10^^-2^) | 12.2 | ± | 0.81 | 12.7 | ± | 0.82 | -0.80 | ± | 1.45 | 11.3 | ± | 0.85 | 12.1 | ± | 0.72 | 0.77 | ± | 0.62 | 0.31 | 0.48 |
| M-VLDL-CE (x10^^-2^) | 7.35 | ± | 0.48 | 8.55 | ± | 0.68 | 1.20 | ± | 0.46 | 6.93 | ± | 0.54 | 7.42 | ± | 0.42 | 0.49 | ± | 0.42 | 0.18 | 0.47 |
| M-VLDL-FC (x10^^-2^) | 4.86 | ± | 0.38 | 4.83 | ± | 0.36 | -0.51 | ± | 0.63 | 4.35 | ± | 0.38 | 4.63 | ± | 0.34 | 0.28 | ± | 0.24 | 0.22 | 0.34 |
| M-VLDL-TG (x10^^-2^) | 25.3 | ± | 1.80 | 24.8 | ± | 1.77 | -2.97 | ± | 3.02 | 23.0 | ± | 1.73 | 24.5 | ± | 1.59 | 1.50 | ± | 1.10 | 0.14 | 0.23 |
| S-VLDL-P (x10^^-10^) | 258 | ± | 10.8 | 278 | ± | 15.9 | 19.8 | ± | 10.3 | 246 | ± | 10.7 | 253 | ± | 10.2 | 6.09 | ± | 6.61 | 0.27 | 0.82 |
| S-VLDL-L (x10^^-2^) | 50.4 | ± | 2.07 | 54.4 | ± | 3.01 | 4.01 | ± | 1.94 | 48.2 | ± | 2.10 | 49.2 | ± | 1.97 | 1.06 | ± | 1.33 | 0.21 | 0.44 |
| S-VLDL-PL (x10^^-2^) | 12.1 | ± | 0.44 | 12.9 | ± | 0.67 | 0.75 | ± | 0.42 | 11.6 | ± | 0.46 | 11.7 | ± | 0.45 | 0.12 | ± | 0.27 | 0.24 | 0.46 |
| S-VLDL-C | 17.3 | ± | 3.88 | 19.3 | ± | 4.32 | 1.98 | ± | 0.69 | 17.0 | ± | 3.80 | 17.2 | ± | 3.85 | 0.19 | ± | 0.78 | 0.04 | 0.08 |
| S-VLDL-CE | 10.6 | ± | 0.61 | 12.1 | ± | 0.64 | 1.47 | ± | 0.50 | 10.6 | ± | 0.77 | 10.7 | ± | 0.55 | 0.12 | ± | 0.64 | 0.04 | 0.07 |
| S-VLDL-FC (x10^^-2^) | 6.74 | ± | 0.28 | 7.26 | ± | 0.40 | 0.51 | ± | 0.26 | 6.42 | ± | 0.29 | 6.49 | ± | 0.28 | 0.07 | ± | 0.19 | 0.17 | 0.32 |
| S-VLDL-TG (x10^^-2^) | 20.9 | ± | 1.03 | 20.4 | ± | 1.02 | -2.57 | ± | 2.10 | 19.6 | ± | 0.99 | 20.3 | ± | 0.97 | 0.76 | ± | 0.59 | 0.14 | 0.25 |
| XS-VLDL-P (x10^^-4^) | 0.35 | ± | 0.08 | 0.36 | ± | 0.08 | 0.02 | ± | 0.01 | 0.34 | ± | 0.08 | 0.33 | ± | 0.07 | -0.01 | ± | 0.01 | 0.04 | 0.31 |
| XS-VLDL-L | 0.44 | ± | 0.10 | 0.46 | ± | 0.10 | 0.02 | ± | 0.01 | 0.43 | ± | 0.10 | 0.42 | ± | 0.09 | -0.01 | ± | 0.01 | 0.04 | 0.04 |
| XS-VLDL-PL (x10^^2^) | 12.9 | ± | 2.88 | 13.5 | ± | 3.01 | 0.58 | ± | 0.39 | 12.6 | ± | 2.81 | 12.1 | ± | 2.71 | -0.42 | ± | 0.43 | 0.06 | 0.03 |
| XS-VLDL-C (x10^^-2^) | 21.9 | ± | 0.75 | 22.6 | ± | 0.64 | -0.40 | ± | 1.53 | 21.7 | ± | 1.04 | 21.0 | ± | 0.84 | -0.71 | ± | 0.91 | 0.85 | 0.94 |
| XS-VLDL-CE (x10^^-2^) | 15.2 | ± | 0.55 | 16.2 | ± | 0.60 | 0.95 | ± | 0.49 | 15.2 | ± | 0.75 | 14.6 | ± | 0.61 | -0.62 | ± | 0.65 | 0.02 | 0.05 |
| XS-VLDL-FC (x10^^-2^) | 6.63 | ± | 0.22 | 6.84 | ± | 0.22 | -0.13 | ± | 0.46 | 6.51 | ± | 0.31 | 6.42 | ± | 0.24 | -0.09 | ± | 0.28 | 0.93 | 0.90 |
| XS-VLDL-TG (x10^^-2^) | 9.34 | ± | 0.30 | 9.34 | ± | 0.34 | -0.47 | ± | 0.59 | 8.88 | ± | 0.27 | 8.98 | ± | 0.31 | 0.10 | ± | 0.20 | 0.40 | 0.29 |
| IDL-P (x10^^-4^) | 0.92 | ± | 0.20 | 0.93 | ± | 0.21 | -0.03 | ± | 0.06 | 0.90 | ± | 0.20 | 0.85 | ± | 0.19 | -0.05 | ± | 0.03 | 0.81 | 0.85 |
| IDL-L (x10^^-2^) | 92.5 | ± | 3.32 | 93.8 | ± | 3.19 | -3.34 | ± | 6.67 | 91.0 | ± | 4.24 | 85.8 | ± | 3.64 | -5.16 | ± | 3.38 | 0.79 | 0.79 |
| IDL-PL (x10^^-2^) | 25.6 | ± | 0.87 | 25.9 | ± | 0.81 | -1.01 | ± | 1.82 | 25.3 | ± | 1.11 | 23.9 | ± | 0.94 | -1.38 | ± | 0.87 | 0.85 | 0.83 |
| IDL-C (x10^^-2^) | 56.9 | ± | 2.35 | 58.0 | ± | 2.22 | -1.75 | ± | 4.38 | 56.1 | ± | 2.99 | 52.7 | ± | 2.55 | -3.45 | ± | 2.43 | 0.72 | 0.73 |
| IDL-CE (x10^^-2^) | 40.6 | ± | 1.68 | 41.6 | ± | 1.59 | -1.04 | ± | 3.11 | 40.0 | ± | 2.12 | 37.6 | ± | 1.83 | -2.36 | ± | 1.74 | 0.69 | 0.73 |
| IDL-FC (x10^^-2^) | 16.3 | ± | 0.70 | 16.4 | ± | 0.67 | -0.71 | ± | 1.27 | 16.1 | ± | 0.89 | 15.0 | ± | 0.74 | -1.09 | ± | 0.70 | 0.78 | 0.72 |
| IDL-TG (x10^^-2^) | 9.95 | ± | 0.28 | 9.91 | ± | 0.32 | -0.04 | ± | 0.26 | 9.56 | ± | 0.27 | 9.23 | ± | 0.28 | -0.33 | ± | 0.22 | 0.45 | 0.34 |
| L-LDL-P (x10^^-7^) | 1.49 | ± | 0.06 | 1.52 | ± | 0.05 | -0.05 | ± | 0.11 | 1.47 | ± | 0.07 | 1.38 | ± | 0.06 | -0.09 | ± | 0.06 | 0.81 | 0.91 |
| L-LDL-L (x10^^-2^) | 107 | ± | 4.03 | 109 | ± | 3.92 | -3.27 | ± | 7.87 | 105 | ± | 5.16 | 98.5 | ± | 4.40 | -6.43 | ± | 4.14 | 0.71 | 0.68 |
| L-LDL-PL (x10^^-2^) | 27.5 | ± | 0.85 | 28.0 | ± | 0.86 | -0.90 | ± | 1.90 | 27.2 | ± | 1.08 | 25.8 | ± | 0.92 | -1.31 | ± | 0.87 | 0.84 | 0.82 |
| L-LDL-C (x10^^-2^) | 70.8 | ± | 3.03 | 72.6 | ± | 2.89 | -1.88 | ± | 5.57 | 69.9 | ± | 3.86 | 65.3 | ± | 3.30 | -4.65 | ± | 3.14 | 0.65 | 0.62 |
| L-LDL-CE (x10^^-2^) | 50.2 | ± | 2.27 | 51.7 | ± | 2.19 | -1.05 | ± | 4.04 | 49.4 | ± | 2.90 | 46.0 | ± | 2.49 | -3.43 | ± | 2.36 | 0.59 | 0.57 |
| L-LDL-FC (x10^^-2^) | 20.6 | ± | 0.77 | 20.8 | ± | 0.72 | -0.83 | ± | 1.54 | 20.5 | ± | 0.97 | 19.3 | ± | 0.82 | -1.22 | ± | 0.78 | 0.81 | 0.75 |
| L-LDL-TG (x10^^-2^) | 8.21 | ± | 0.28 | 8.21 | ± | 0.31 | -0.01 | ± | 0.23 | 7.88 | ± | 0.30 | 7.41 | ± | 0.27 | -0.47 | ± | 0.21 | 0.18 | 0.11 |
| M-LDL-P (x10^^-4^) | 1.20 | ± | 0.27 | 1.27 | ± | 0.28 | 0.07 | ± | 0.04 | 1.18 | ± | 0.26 | 1.11 | ± | 0.25 | -0.08 | ± | 0.05 | 0.02 | 0.15 |
| M-LDL-L | 0.61 | ± | 0.14 | 0.65 | ± | 0.14 | 0.03 | ± | 0.02 | 0.60 | ± | 0.14 | 0.57 | ± | 0.13 | -0.04 | ± | 0.02 | 0.02 | 0.01 |
| M-LDL-PL | 17.3 | ± | 0.48 | 18.0 | ± | 0.64 | 0.73 | ± | 0.40 | 16.9 | ± | 0.57 | 16.2 | ± | 0.53 | -0.75 | ± | 0.43 | 0.01 | 0.01 |
| M-LDL-C (x10^^-2^) | 40.0 | ± | 1.84 | 41.1 | ± | 1.83 | -1.01 | ± | 3.25 | 39.6 | ± | 2.30 | 36.7 | ± | 1.99 | -2.87 | ± | 1.88 | 0.60 | 0.53 |
| M-LDL-CE (x10^^-2^) | 27.9 | ± | 1.49 | 28.8 | ± | 1.47 | -0.54 | ± | 2.45 | 27.6 | ± | 1.88 | 25.4 | ± | 1.61 | -2.26 | ± | 1.54 | 0.53 | 0.45 |
| M-LDL-FC (x10^^-2^) | 12.1 | ± | 0.35 | 12.2 | ± | 0.36 | -0.47 | ± | 0.81 | 12.0 | ± | 0.42 | 11.4 | ± | 0.38 | -0.60 | ± | 0.33 | 0.88 | 0.83 |
| M-LDL-TG (x10^^-2^) | 4.08 | ± | 0.16 | 4.07 | ± | 0.17 | -0.02 | ± | 0.12 | 3.88 | ± | 0.18 | 3.61 | ± | 0.16 | -0.27 | ± | 0.11 | 0.17 | 0.11 |
| S-LDL-P (x10^^-4^) | 1.40 | ± | 0.31 | 1.47 | ± | 0.33 | 0.07 | ± | 0.04 | 1.37 | ± | 0.31 | 1.28 | ± | 0.29 | -0.09 | ± | 0.05 | 0.01 | 0.13 |
| S-LDL-L | 0.39 | ± | 0.09 | 0.41 | ± | 0.09 | 0.02 | ± | 0.01 | 0.39 | ± | 0.09 | 0.36 | ± | 0.08 | -0.03 | ± | 0.01 | 0.01 | 0.01 |
| S-LDL-PL (x10^^2^) | 12.5 | ± | 2.78 | 12.9 | ± | 2.87 | 0.42 | ± | 0.25 | 12.2 | ± | 2.72 | 11.6 | ± | 2.60 | -0.55 | ± | 0.25 | 0.01 | 0.01 |
| S-LDL-C (x10^^-2^) | 24.4 | ± | 1.11 | 24.9 | ± | 1.14 | -0.73 | ± | 1.96 | 24.1 | ± | 1.36 | 22.3 | ± | 1.20 | -1.83 | ± | 1.09 | 0.61 | 0.52 |
| S-LDL-CE (x10^^-2^) | 17.2 | ± | 0.90 | 17.6 | ± | 0.91 | -0.44 | ± | 1.48 | 17.0 | ± | 1.11 | 15.5 | ± | 0.97 | -1.44 | ± | 0.89 | 0.55 | 0.45 |
| S-LDL-FC (x10^2) | 7.23 | ± | 1.61 | 7.48 | ± | 1.67 | 0.25 | ± | 0.17 | 7.14 | ± | 1.60 | 6.75 | ± | 1.51 | -0.40 | ± | 0.20 | 0.02 | 0.01 |
| S-LDL-TG (x10^^-2^) | 2.53 | ± | 0.10 | 2.61 | ± | 0.13 | 0.08 | ± | 0.09 | 2.35 | ± | 0.12 | 2.22 | ± | 0.10 | -0.13 | ± | 0.07 | 0.14 | 0.18 |
| XL-HDL-P (x10^^-7^) | 3.45 | ± | 0.34 | 3.36 | ± | 0.33 | -0.09 | ± | 0.15 | 3.16 | ± | 0.31 | 2.94 | ± | 0.28 | -0.22 | ± | 0.12 | 0.37 | 0.46 |
| XL-HDL-L (x10^^-2^) | 35.1 | ± | 3.43 | 34.1 | ± | 3.31 | -0.97 | ± | 1.54 | 32.2 | ± | 3.17 | 29.9 | ± | 2.83 | -2.29 | ± | 1.21 | 0.37 | 0.26 |
| XL-HDL-PL (x10^^-2^) | 15.9 | ± | 1.89 | 15.1 | ± | 1.89 | -0.77 | ± | 0.89 | 14.4 | ± | 1.74 | 13.8 | ± | 1.51 | -0.59 | ± | 0.65 | 0.82 | 0.67 |
| XL-HDL-C (x10^^-2^) | 18.2 | ± | 1.53 | 17.9 | ± | 1.45 | -0.34 | ± | 0.70 | 16.8 | ± | 1.49 | 15.2 | ± | 1.33 | -1.65 | ± | 0.66 | 0.15 | 0.18 |
| XL-HDL-CE (x10^^-2^) | 14.0 | ± | 1.07 | 13.8 | ± | 1.01 | -0.18 | ± | 0.51 | 13.0 | ± | 1.09 | 11.7 | ± | 0.96 | -1.34 | ± | 0.52 | 0.12 | 0.15 |
| XL-HDL-FC (x10^^-2^) | 4.23 | ± | 0.46 | 4.07 | ± | 0.45 | -0.17 | ± | 0.21 | 3.84 | ± | 0.41 | 3.53 | ± | 0.38 | -0.31 | ± | 0.15 | 0.45 | 0.43 |
| XL-HDL-TG (x10^^-2^) | 0.99 | ± | 0.07 | 1.14 | ± | 0.09 | 0.15 | ± | 0.08 | 0.90 | ± | 0.10 | 0.85 | ± | 0.07 | -0.04 | ± | 0.08 | 0.13 | 0.27 |
| L-HDL-P (x10^^-7^) | 6.05 | ± | 0.83 | 5.62 | ± | 0.68 | -0.97 | ± | 0.80 | 5.97 | ± | 0.84 | 5.42 | ± | 0.68 | -0.23 | ± | 0.41 | 0.39 | 0.03 |
| L-HDL-L (x10^^-2^) | 37.8 | ± | 5.29 | 34.9 | ± | 4.36 | -6.28 | ± | 5.04 | 35.4 | ± | 5.52 | 33.8 | ± | 4.33 | -1.50 | ± | 2.54 | 0.28 | 0.60 |
| L-HDL-PL (x10^^-2^) | 18.6 | ± | 2.42 | 19.1 | ± | 2.49 | -0.43 | ± | 1.25 | 17.5 | ± | 2.65 | 16.6 | ± | 2.00 | -0.82 | ± | 1.33 | 0.80 | 0.54 |
| L-HDL-C (x10^^-2^) | 18.6 | ± | 2.63 | 15.6 | ± | 2.21 | -3.50 | ± | 2.50 | 17.4 | ± | 2.63 | 15.7 | ± | 2.22 | -0.74 | ± | 1.09 | 0.30 | 0.55 |
| L-HDL-CE (x10^^-2^) | 14.1 | ± | 2.09 | 11.8 | ± | 1.77 | -2.73 | ± | 1.94 | 13.8 | ± | 2.04 | 12.6 | ± | 1.70 | -0.48 | ± | 0.86 | 0.28 | 0.50 |
| L-HDL-FC (x10^^-2^) | 3.58 | ± | 0.64 | 3.54 | ± | 0.67 | -0.21 | ± | 0.29 | 3.59 | ± | 0.60 | 3.13 | ± | 0.52 | -0.26 | ± | 0.23 | 0.89 | 0.33 |
| L-HDL-TG (x10^^-2^) | 1.61 | ± | 0.20 | 1.89 | ± | 0.20 | 0.17 | ± | 0.13 | 1.55 | ± | 0.27 | 1.53 | ± | 0.16 | 0.06 | ± | 0.18 | 0.57 | 0.69 |
| M-HDL-P (x10^^-7^) | 13.5 | ± | 0.60 | 13.8 | ± | 0.62 | 0.32 | ± | 0.30 | 13.4 | ± | 0.66 | 13.0 | ± | 0.56 | -0.35 | ± | 0.47 | 0.21 | 0.21 |
| M-HDL-L (x10^^-2^) | 56.3 | ± | 2.61 | 57.6 | ± | 2.68 | 1.27 | ± | 1.28 | 55.9 | ± | 2.86 | 54.4 | ± | 2.45 | -1.57 | ± | 2.04 | 0.21 | 0.19 |
| M-HDL-PL (x10^^-2^) | 27.6 | ± | 1.22 | 27.9 | ± | 1.23 | 0.37 | ± | 0.58 | 27.3 | ± | 1.38 | 26.2 | ± | 1.14 | -1.09 | ± | 0.91 | 0.15 | 0.15 |
| M-HDL-C (x10^^-2^) | 25.2 | ± | 1.39 | 25.9 | ± | 1.42 | 0.62 | ± | 0.69 | 25.3 | ± | 1.48 | 24.7 | ± | 1.29 | -0.63 | ± | 1.09 | 0.32 | 0.21 |
| M-HDL-CE (x10^^-2^) | 21.0 | ± | 1.08 | 21.6 | ± | 1.10 | 0.53 | ± | 0.55 | 21.2 | ± | 1.13 | 20.8 | ± | 1.00 | -0.39 | ± | 0.83 | 0.34 | 0.23 |
| M-HDL-FC (x10^^-2^) | 4.21 | ± | 0.31 | 4.29 | ± | 0.32 | 0.08 | ± | 0.15 | 4.18 | ± | 0.36 | 3.95 | ± | 0.30 | -0.23 | ± | 0.26 | 0.25 | 0.16 |
| M-HDL-TG (x10^^-2^) | 3.49 | ± | 0.13 | 3.77 | ± | 0.20 | 0.29 | ± | 0.14 | 3.34 | ± | 0.16 | 3.49 | ± | 0.13 | 0.15 | ± | 0.11 | 0.09 | 0.37 |
| S-HDL-P (x10^^-7^) | 42.8 | ± | 0.86 | 43.1 | ± | 0.91 | 0.31 | ± | 0.51 | 42.9 | ± | 0.85 | 42.0 | ± | 0.92 | -0.83 | ± | 0.67 | 0.13 | 0.23 |
| S-HDL-L (x10^^-2^) | 94.5 | ± | 1.90 | 95.1 | ± | 2.00 | 0.67 | ± | 1.13 | 94.8 | ± | 1.90 | 92.9 | ± | 2.06 | -1.92 | ± | 1.53 | 0.12 | 0.09 |
| S-HDL-PL (x10^^-2^) | 49.0 | ± | 1.22 | 49.3 | ± | 1.24 | 0.30 | ± | 0.78 | 48.9 | ± | 1.18 | 48.2 | ± | 1.17 | -0.61 | ± | 0.72 | 0.39 | 0.63 |
| S-HDL-C (x10^^-2^) | 40.4 | ± | 0.84 | 40.7 | ± | 1.01 | 0.25 | ± | 0.78 | 41.0 | ± | 0.93 | 39.7 | ± | 1.03 | -1.34 | ± | 0.92 | 0.18 | 0.04 |
| S-HDL-CE (x10^^-2^) | 31.8 | ± | 0.70 | 32.0 | ± | 0.87 | 0.21 | ± | 0.75 | 32.4 | ± | 0.79 | 31.2 | ± | 0.86 | -1.21 | ± | 0.79 | 0.20 | 0.05 |
| S-HDL-FC (x10^^-2^) | 8.63 | ± | 0.23 | 8.68 | ± | 0.23 | 0.04 | ± | 0.13 | 8.65 | ± | 0.22 | 8.51 | ± | 0.24 | -0.13 | ± | 0.17 | 0.40 | 0.37 |
| S-HDL-TG (x10^^-2^) | 5.07 | ± | 0.15 | 5.01 | ± | 0.17 | -0.31 | ± | 0.30 | 4.91 | ± | 0.17 | 4.83 | ± | 0.11 | -0.32 | ± | 0.36 | 0.92 | 0.60 |
| **Relative lipoprotein lipid concentrations** | | | | |  |  |  |  |  |  |  |  |  |  |  |  |  |  |  |  |
| XXL-VLDL-PL_% | 9.78 | ± | 0.36 | 9.74 | ± | 0.38 | -0.04 | ± | 0.40 | 9.16 | ± | 0.48 | 8.86 | ± | 0.46 | 0.18 | ± | 0.41 | 0.70 | 0.19 |
| XXL-VLDL-C_% | 14.4 | ± | 0.50 | 15.3 | ± | 0.42 | 0.83 | ± | 0.81 | 14.7 | ± | 0.71 | 15.2 | ± | 0.46 | 1.94 | ± | 1.08 | 0.27 | 0.22 |
| XXL-VLDL-CE_% | 9.11 | ± | 0.72 | 9.86 | ± | 0.66 | 0.71 | ± | 0.42 | 9.80 | ± | 0.97 | 10.9 | ± | 0.59 | 1.46 | ± | 0.90 | 0.42 | 0.71 |
| XXL-VLDL-FC_% | 4.76 | ± | 0.28 | 4.81 | ± | 0.29 | 0.05 | ± | 0.29 | 4.27 | ± | 0.29 | 4.36 | ± | 0.31 | 0.30 | ± | 0.25 | 0.70 | 0.20 |
| XXL-VLDL-TG_% | 76.3 | ± | 0.61 | 74.8 | ± | 0.40 | -5.23 | ± | 4.01 | 76.8 | ± | 0.88 | 75.9 | ± | 0.57 | 3.05 | ± | 4.14 | 0.16 | 0.02 |
| XL-VLDL-PL_% | 13.3 | ± | 0.57 | 14.8 | ± | 0.38 | 0.65 | ± | 0.74 | 13.6 | ± | 0.67 | 13.8 | ± | 0.47 | 0.20 | ± | 1.10 | 0.72 | 0.83 |
| XL-VLDL-C_% | 22.6 | ± | 1.29 | 23.6 | ± | 1.03 | -0.26 | ± | 1.04 | 24.4 | ± | 1.10 | 24.8 | ± | 1.06 | 2.84 | ± | 2.11 | 0.21 | 0.13 |
| XL-VLDL-CE_% | 15.3 | ± | 1.21 | 15.3 | ± | 0.91 | -0.81 | ± | 0.88 | 16.7 | ± | 1.03 | 16.8 | ± | 0.97 | 0.93 | ± | 1.04 | 0.20 | 0.17 |
| XL-VLDL-FC_% | 7.30 | ± | 0.39 | 8.32 | ± | 0.31 | 0.56 | ± | 0.43 | 7.67 | ± | 0.51 | 7.33 | ± | 0.43 | 0.45 | ± | 0.67 | 0.88 | 0.57 |
| XL-VLDL-TG_% | 63.3 | ± | 1.09 | 62.6 | ± | 0.61 | -3.76 | ± | 5.75 | 63.0 | ± | 1.13 | 62.2 | ± | 1.04 | 8.76 | ± | 4.85 | 0.07 | 0.01 |
| L-VLDL-PL_% | 17.1 | ± | 0.14 | 17.4 | ± | 0.13 | 0.24 | ± | 0.14 | 17.2 | ± | 0.19 | 17.0 | ± | 0.15 | 0.72 | ± | 0.80 | 0.56 | 0.04 |
| L-VLDL-C_% | 23.3 | ± | 0.63 | 24.2 | ± | 0.52 | -0.32 | ± | 1.02 | 24.1 | ± | 0.56 | 23.9 | ± | 0.54 | 2.23 | ± | 1.48 | 0.22 | 0.02 |
| L-VLDL-CE_% | 15.7 | ± | 0.81 | 15.5 | ± | 0.79 | -0.12 | ± | 0.44 | 16.6 | ± | 0.83 | 16.8 | ± | 0.66 | 0.98 | ± | 0.84 | 0.29 | 0.06 |
| L-VLDL-FC_% | 7.62 | ± | 0.38 | 8.29 | ± | 0.31 | 0.22 | ± | 0.51 | 6.98 | ± | 0.51 | 7.17 | ± | 0.39 | 0.53 | ± | 0.42 | 0.69 | 0.11 |
| L-VLDL-TG_% | 59.1 | ± | 0.50 | 58.8 | ± | 0.35 | -3.25 | ± | 5.29 | 58.6 | ± | 0.66 | 58.6 | ± | 0.48 | 2.91 | ± | 3.01 | 0.16 | 0.01 |
| M-VLDL-PL_% | 20.3 | ± | 0.09 | 20.3 | ± | 0.12 | 0.02 | ± | 0.10 | 20.4 | ± | 0.10 | 20.2 | ± | 0.08 | -0.23 | ± | 0.09 | 0.06 | 0.01 |
| M-VLDL-C_% | 26.2 | ± | 0.75 | 27.0 | ± | 0.77 | 0.80 | ± | 0.43 | 26.1 | ± | 0.87 | 26.4 | ± | 0.60 | 0.31 | ± | 0.68 | 0.35 | 0.39 |
| M-VLDL-CE_% | 16.0 | ± | 0.84 | 16.7 | ± | 0.86 | 0.72 | ± | 0.42 | 16.2 | ± | 0.86 | 16.5 | ± | 0.63 | 0.24 | ± | 0.67 | 0.40 | 0.33 |
| M-VLDL-FC_% | 10.2 | ± | 0.16 | 10.3 | ± | 0.18 | 0.08 | ± | 0.14 | 9.90 | ± | 0.18 | 9.97 | ± | 0.16 | 0.07 | ± | 0.12 | 0.96 | 0.62 |
| M-VLDL-TG_% | 53.5 | ± | 0.79 | 52.7 | ± | 0.82 | -0.82 | ± | 0.45 | 53.4 | ± | 0.92 | 53.4 | ± | 0.65 | -0.08 | ± | 0.72 | 0.23 | 0.20 |
| S-VLDL-PL_% | 24.2 | ± | 0.26 | 23.8 | ± | 0.20 | -0.41 | ± | 0.17 | 24.2 | ± | 0.30 | 23.9 | ± | 0.22 | -0.36 | ± | 0.22 | 0.77 | 0.94 |
| S-VLDL-C_% | 34.5 | ± | 0.97 | 35.3 | ± | 0.74 | -0.94 | ± | 2.31 | 35.1 | ± | 1.12 | 35.0 | ± | 0.84 | -0.16 | ± | 0.94 | 0.75 | 0.80 |
| S-VLDL-CE_% | 21.1 | ± | 0.97 | 22.0 | ± | 0.71 | -0.21 | ± | 1.69 | 21.8 | ± | 1.12 | 21.8 | ± | 0.84 | 0.02 | ± | 0.94 | 0.90 | 0.96 |
| S-VLDL-FC_% | 13.4 | ± | 0.12 | 13.4 | ± | 0.12 | -0.04 | ± | 0.06 | 13.3 | ± | 0.12 | 13.1 | ± | 0.13 | -0.18 | ± | 0.10 | 0.21 | 0.11 |
| S-VLDL-TG_% | 41.3 | ± | 0.89 | 40.3 | ± | 0.96 | -1.03 | ± | 0.72 | 40.7 | ± | 0.95 | 41.2 | ± | 0.79 | 0.52 | ± | 0.78 | 0.13 | 0.07 |
| XS-VLDL-PL_% | 29.1 | ± | 0.32 | 29.0 | ± | 0.37 | -0.14 | ± | 0.31 | 29.0 | ± | 0.39 | 28.7 | ± | 0.27 | -0.31 | ± | 0.30 | 0.71 | 0.17 |
| XS-VLDL-C_% | 49.5 | ± | 0.60 | 50.1 | ± | 0.56 | 0.66 | ± | 0.57 | 50.1 | ± | 0.62 | 49.7 | ± | 0.64 | -0.34 | ± | 0.64 | 0.24 | 0.27 |
| XS-VLDL-CE_% | 34.5 | ± | 0.54 | 35.0 | ± | 0.45 | 0.52 | ± | 0.53 | 35.1 | ± | 0.52 | 34.5 | ± | 0.59 | -0.52 | ± | 0.56 | 0.19 | 0.41 |
| XS-VLDL-FC_% | 15.0 | ± | 0.15 | 15.2 | ± | 0.20 | 0.14 | ± | 0.18 | 15.0 | ± | 0.24 | 15.2 | ± | 0.17 | 0.19 | ± | 0.22 | 0.87 | 0.31 |
| XS-VLDL-TG_% | 21.4 | ± | 0.74 | 20.9 | ± | 0.78 | -0.52 | ± | 0.62 | 20.9 | ± | 0.73 | 21.5 | ± | 0.75 | 0.65 | ± | 0.64 | 0.21 | 0.10 |
| IDL-PL_% | 27.8 | ± | 6.21 | 27.7 | ± | 6.18 | -0.12 | ± | 0.09 | 27.9 | ± | 6.23 | 27.9 | ± | 6.25 | 0.08 | ± | 0.11 | 0.11 | 0.15 |
| IDL-C_% | 61.6 | ± | 13.8 | 61.9 | ± | 13.8 | 3.37 | ± | 2.96 | 61.4 | ± | 13.7 | 61.1 | ± | 13.7 | -0.30 | ± | 0.43 | 0.25 | 0.01 |
| IDL-CE_% | 43.7 | ± | 9.78 | 44.4 | ± | 9.93 | 0.67 | ± | 0.22 | 43.7 | ± | 9.78 | 43.6 | ± | 9.75 | -0.12 | ± | 0.36 | 0.04 | 0.04 |
| IDL-FC_% | 17.6 | ± | 0.17 | 17.5 | ± | 0.25 | -0.04 | ± | 0.19 | 17.6 | ± | 0.22 | 17.5 | ± | 0.18 | -0.17 | ± | 0.16 | 0.58 | 0.19 |
| IDL-TG_% | 11.0 | ± | 0.43 | 10.5 | ± | 0.38 | -0.50 | ± | 0.34 | 10.8 | ± | 0.38 | 11.0 | ± | 0.40 | 0.22 | ± | 0.37 | 0.19 | 0.09 |
| L-LDL-PL_% | 26.0 | ± | 0.19 | 25.8 | ± | 0.19 | -0.19 | ± | 0.16 | 26.1 | ± | 0.22 | 26.4 | ± | 0.25 | 0.36 | ± | 0.21 | 0.02 | 0.001 |
| L-LDL-C_% | 66.2 | ± | 0.43 | 66.8 | ± | 0.37 | 0.59 | ± | 0.36 | 66.3 | ± | 0.40 | 65.9 | ± | 0.43 | -0.36 | ± | 0.40 | 0.08 | 0.02 |
| L-LDL-CE_% | 46.8 | ± | 0.44 | 47.6 | ± | 0.37 | 0.76 | ± | 0.33 | 46.7 | ± | 0.44 | 46.3 | ± | 0.48 | -0.41 | ± | 0.43 | 0.02 | 0.01 |
| L-LDL-FC_% | 19.4 | ± | 0.11 | 19.3 | ± | 0.13 | -1.01 | ± | 0.95 | 19.6 | ± | 0.14 | 19.6 | ± | 0.13 | 0.05 | ± | 0.09 | 0.28 | 0.25 |
| L-LDL-TG_% | 7.82 | ± | 0.30 | 7.43 | ± | 0.25 | -0.40 | ± | 0.24 | 7.64 | ± | 0.24 | 7.64 | ± | 0.24 | 0.001 | ± | 0.24 | 0.29 | 0.16 |
| M-LDL-PL_% | 28.4 | ± | 6.34 | 28.1 | ± | 6.28 | -0.29 | ± | 0.36 | 28.4 | ± | 6.35 | 29.0 | ± | 6.48 | 0.57 | ± | 0.43 | 0.11 | 0.02 |
| M-LDL-C_% | 64.9 | ± | 0.56 | 65.5 | ± | 0.58 | 0.66 | ± | 0.57 | 65.1 | ± | 0.60 | 64.6 | ± | 0.56 | -0.51 | ± | 0.60 | 0.16 | 0.04 |
| M-LDL-CE_% | 45.0 | ± | 10.1 | 46.0 | ± | 10.3 | 1.00 | ± | 0.70 | 45.0 | ± | 10.1 | 44.2 | ± | 9.89 | -0.81 | ± | 0.82 | 0.08 | 0.02 |
| M-LDL-FC_% | 19.8 | ± | 4.43 | 19.5 | ± | 4.36 | -0.34 | ± | 0.17 | 20.0 | ± | 4.48 | 20.3 | ± | 4.55 | 0.30 | ± | 0.24 | 0.02 | 0.002 |
| M-LDL-TG_% | 6.75 | ± | 0.27 | 6.38 | ± | 0.23 | -0.37 | ± | 0.23 | 6.52 | ± | 0.23 | 6.46 | ± | 0.22 | -0.06 | ± | 0.23 | 0.39 | 0.23 |
| S-LDL-PL_% | 31.9 | ± | 7.12 | 31.4 | ± | 7.03 | -0.44 | ± | 0.44 | 31.9 | ± | 7.13 | 32.6 | ± | 7.28 | 0.65 | ± | 0.50 | 0.08 | 0.01 |
| S-LDL-C_% | 61.6 | ± | 0.63 | 62.2 | ± | 0.68 | 0.55 | ± | 0.68 | 61.9 | ± | 0.72 | 61.2 | ± | 0.65 | -0.70 | ± | 0.68 | 0.20 | 0.05 |
| S-LDL-CE_% | 43.2 | ± | 9.68 | 44.0 | ± | 9.85 | 0.81 | ± | 0.76 | 43.3 | ± | 9.68 | 42.4 | ± | 9.49 | -0.89 | ± | 0.80 | 0.12 | 0.02 |
| S-LDL-FC_% | 18.5 | ± | 4.12 | 18.2 | ± | 4.07 | -0.26 | ± | 0.14 | 18.6 | ± | 4.17 | 18.8 | ± | 4.21 | 0.19 | ± | 0.15 | 0.02 | 0.001 |
| S-LDL-TG_% | 6.49 | ± | 0.24 | 6.18 | ± | 0.19 | -0.62 | ± | 0.37 | 6.16 | ± | 0.25 | 6.20 | ± | 0.20 | 0.05 | ± | 0.23 | 0.12 | 0.02 |
| XL-HDL-PL_% | 43.6 | ± | 9.82 | 43.5 | ± | 9.75 | -2.28 | ± | 1.88 | 44.5 | ± | 9.94 | 46.3 | ± | 10.4 | 1.76 | ± | 0.97 | 0.05 | 0.10 |
| XL-HDL-C_% | 53.3 | ± | 1.12 | 53.1 | ± | 1.28 | -2.86 | ± | 3.19 | 52.4 | ± | 1.30 | 50.8 | ± | 0.99 | -1.52 | ± | 0.77 | 0.69 | 0.45 |
| XL-HDL-CE_% | 41.5 | ± | 9.20 | 42.4 | ± | 9.44 | 0.93 | ± | 0.91 | 40.6 | ± | 9.08 | 39.1 | ± | 8.75 | -1.43 | ± | 0.74 | 0.03 | 0.08 |
| XL-HDL-FC_% | 12.0 | ± | 0.18 | 11.6 | ± | 0.28 | 0.25 | ± | 0.55 | 11.8 | ± | 0.24 | 11.7 | ± | 0.23 | -0.14 | ± | 0.75 | 0.66 | 0.83 |
| XL-HDL-TG_% | 3.14 | ± | 0.28 | 3.45 | ± | 0.27 | 0.14 | ± | 0.24 | 2.91 | ± | 0.38 | 2.88 | ± | 0.21 | -0.03 | ± | 0.51 | 0.69 | 0.89 |
| L-HDL-PL_% | 50.4 | ± | 0.93 | 51.2 | ± | 0.93 | 0.73 | ± | 4.30 | 49.9 | ± | 0.55 | 50.0 | ± | 0.68 | 7.58 | ± | 4.37 | 0.18 | 0.21 |
| L-HDL-C_% | 44.9 | ± | 10.0 | 43.2 | ± | 9.63 | -1.52 | ± | 2.67 | 45.8 | ± | 10.2 | 45.0 | ± | 10.1 | 6.15 | ± | 3.55 | 0.03 | 0.12 |
| L-HDL-CE_% | 36.4 | ± | 8.11 | 35.2 | ± | 7.85 | -1.03 | ± | 2.31 | 36.6 | ± | 8.19 | 36.7 | ± | 8.21 | 5.59 | ± | 3.06 | 0.02 | 0.09 |
| L-HDL-FC_% | 8.53 | ± | 0.50 | 7.99 | ± | 0.59 | -0.49 | ± | 0.46 | 9.14 | ± | 0.34 | 8.29 | ± | 0.48 | 0.56 | ± | 0.54 | 0.09 | 0.47 |
| L-HDL-TG_% | 4.69 | ± | 0.39 | 5.56 | ± | 0.46 | 0.79 | ± | 0.62 | 4.33 | ± | 0.53 | 4.98 | ± | 0.41 | 1.27 | ± | 0.43 | 0.41 | 0.11 |
| M-HDL-PL_% | 49.1 | ± | 0.31 | 48.6 | ± | 0.29 | -0.43 | ± | 0.22 | 48.8 | ± | 0.29 | 48.2 | ± | 0.23 | -0.59 | ± | 0.29 | 0.71 | 0.84 |
| M-HDL-C_% | 44.6 | ± | 0.41 | 44.7 | ± | 0.46 | 0.09 | ± | 0.34 | 45.0 | ± | 0.60 | 45.2 | ± | 0.46 | 0.22 | ± | 0.44 | 0.84 | 0.46 |
| M-HDL-CE_% | 37.3 | ± | 0.33 | 37.4 | ± | 0.34 | 0.10 | ± | 0.30 | 37.8 | ± | 0.44 | 38.1 | ± | 0.36 | 0.37 | ± | 0.30 | 0.60 | 0.70 |
| M-HDL-FC_% | 7.31 | ± | 0.21 | 7.29 | ± | 0.21 | -0.02 | ± | 0.10 | 7.23 | ± | 0.29 | 7.08 | ± | 0.23 | -0.15 | ± | 0.23 | 0.57 | 0.27 |
| M-HDL-TG_% | 6.36 | ± | 0.28 | 6.70 | ± | 0.35 | 0.34 | ± | 0.19 | 6.22 | ± | 0.39 | 6.59 | ± | 0.30 | 0.37 | ± | 0.24 | 0.91 | 0.20 |
| S-HDL-PL_% | 51.8 | ± | 0.52 | 51.8 | ± | 0.59 | -0.01 | ± | 0.53 | 51.5 | ± | 0.52 | 52.0 | ± | 0.56 | 0.44 | ± | 0.35 | 0.54 | 0.15 |
| S-HDL-C_% | 42.9 | ± | 0.53 | 42.8 | ± | 0.69 | -0.05 | ± | 0.60 | 43.3 | ± | 0.57 | 42.7 | ± | 0.57 | -0.57 | ± | 0.44 | 0.55 | 0.14 |
| S-HDL-CE_% | 33.7 | ± | 0.60 | 33.7 | ± | 0.73 | -0.03 | ± | 0.66 | 34.2 | ± | 0.61 | 33.6 | ± | 0.63 | -0.61 | ± | 0.45 | 0.53 | 0.15 |
| S-HDL-FC_% | 9.12 | ± | 0.09 | 9.10 | ± | 0.08 | -0.02 | ± | 0.07 | 9.11 | ± | 0.08 | 9.15 | ± | 0.09 | 0.04 | ± | 0.05 | 0.55 | 0.46 |
| S-HDL-TG_% | 5.37 | ± | 0.13 | 5.43 | ± | 0.19 | 0.06 | ± | 0.13 | 5.20 | ± | 0.17 | 5.34 | ± | 0.13 | 0.13 | ± | 0.12 | 0.70 | 0.29 |
| VLDL-D (nm) | 36.7 | ± | 0.19 | 36.8 | ± | 0.25 | 0.13 | ± | 0.18 | 36.4 | ± | 0.20 | 36.7 | ± | 0.17 | 0.24 | ± | 0.14 | 0.68 | 0.26 |
| LDL-D (nm) | 23.6 | ± | 0.02 | 23.6 | ± | 0.02 | 0.001 | ± | 0.01 | 23.6 | ± | 0.02 | 23.6 | ± | 0.02 | 0.01 | ± | 0.01 | 0.26 | 0.23 |
| HDL-D (nm) | 9.73 | ± | 0.06 | 9.72 | ± | 0.05 | -0.01 | ± | 0.03 | 9.69 | ± | 0.05 | 9.68 | ± | 0.05 | -0.01 | ± | 0.02 | 0.93 | 0.61 |
| **Cholesterol** |  |  |  |  |  |  |  |  |  |  |  |  |  |  |  |  |  |  |  |  |
| Serum-C | 3.51 | ± | 0.78 | 3.69 | ± | 0.82 | 0.18 | ± | 0.09 | 3.44 | ± | 0.77 | 3.28 | ± | 0.73 | -0.17 | ± | 0.12 | 0.01 | 0.01 |
| VLDL-C | 0.58 | ± | 0.13 | 0.64 | ± | 0.14 | 0.07 | ± | 0.02 | 0.55 | ± | 0.12 | 0.56 | ± | 0.13 | 0.01 | ± | 0.02 | 0.05 | 0.16 |
| Remnant-C | 1.14 | ± | 0.26 | 1.24 | ± | 0.28 | 0.10 | ± | 0.04 | 1.11 | ± | 0.25 | 1.09 | ± | 0.24 | -0.03 | ± | 0.04 | 0.01 | 0.02 |
| LDL-C | 1.35 | ± | 0.30 | 1.39 | ± | 0.31 | -0.04 | ± | 0.11 | 1.34 | ± | 0.30 | 1.24 | ± | 0.28 | -0.09 | ± | 0.06 | 0.62 | 0.57 |
| HDL-C | 1.01 | ± | 0.22 | 1.00 | ± | 0.22 | 0.01 | ± | 0.02 | 0.99 | ± | 0.22 | 0.95 | ± | 0.21 | -0.05 | ± | 0.03 | 0.22 | 0.06 |
| HDL2-C | 0.55 | ± | 0.12 | 0.51 | ± | 0.11 | -0.06 | ± | 0.05 | 0.53 | ± | 0.12 | 0.50 | ± | 0.11 | -0.03 | ± | 0.02 | 0.61 | 0.91 |
| HDL3-C (x10^^2^) | 46.22 | ± | 10.3 | 46.24 | ± | 10.3 | 0.02 | ± | 0.25 | 46.22 | ± | 10.3 | 44.9 | ± | 10.0 | -1.31 | ± | 0.39 | 0.01 | 0.01 |
| EstC | 2.43 | ± | 0.54 | 2.50 | ± | 0.56 | -0.06 | ± | 0.17 | 2.38 | ± | 0.53 | 2.27 | ± | 0.51 | -0.12 | ± | 0.09 | 0.73 | 0.72 |
| FreeC | 1.08 | ± | 0.24 | 1.13 | ± | 0.25 | 0.05 | ± | 0.02 | 1.06 | ± | 0.24 | 1.01 | ± | 0.23 | -0.05 | ± | 0.03 | 0.01 | 0.01 |
| **Glycerides and phospholipids** | | | |  |  |  |  |  |  |  |  |  |  |  |  |  |  |  |  |  |
| Serum-TG | 1.09 | ± | 0.24 | 1.07 | ± | 0.24 | -0.13 | ± | 0.11 | 1.00 | ± | 0.22 | 1.03 | ± | 0.23 | 0.02 | ± | 0.03 | 0.19 | 0.32 |
| VLDL-TG | 0.73 | ± | 0.16 | 0.71 | ± | 0.16 | -0.09 | ± | 0.08 | 0.66 | ± | 0.15 | 0.69 | ± | 0.16 | 0.03 | ± | 0.03 | 0.15 | 0.24 |
| LDL-TG (x10^^2^) | 14.8 | ± | 3.30 | 14.9 | ± | 3.32 | 0.06 | ± | 0.44 | 14.1 | ± | 3.15 | 13.2 | ± | 2.96 | -0.87 | ± | 0.38 | 0.16 | 0.12 |
| HDL-TG (x10^^2^) | 11.2 | ± | 2.50 | 11.9 | ± | 2.66 | 0.70 | ± | 0.41 | 10.7 | ± | 2.39 | 10.8 | ± | 2.41 | 0.12 | ± | 0.38 | 0.29 | 0.56 |
| TotPG | 1.42 | ± | 0.32 | 1.47 | ± | 0.33 | 0.05 | ± | 0.04 | 1.35 | ± | 0.30 | 1.34 | ± | 0.30 | -0.02 | ± | 0.04 | 0.22 | 0.21 |
| TG/PG | 0.55 | ± | 0.04 | 0.59 | ± | 0.05 | 0.04 | ± | 0.03 | 0.52 | ± | 0.04 | 0.56 | ± | 0.03 | 0.04 | ± | 0.03 | 0.94 | 0.66 |
| PC | 1.36 | ± | 0.05 | 1.42 | ± | 0.05 | 0.06 | ± | 0.03 | 1.30 | ± | 0.05 | 1.29 | ± | 0.04 | -0.01 | ± | 0.04 | 0.19 | 0.14 |
| SM | 0.43 | ± | 0.10 | 0.45 | ± | 0.10 | 0.01 | ± | 0.01 | 0.43 | ± | 0.10 | 0.41 | ± | 0.09 | -0.02 | ± | 0.01 | 0.03 | 0.01 |
| TotCho | 1.79 | ± | 0.40 | 1.84 | ± | 0.41 | 0.05 | ± | 0.04 | 1.73 | ± | 0.39 | 1.70 | ± | 0.38 | -0.03 | ± | 0.04 | 0.18 | 0.12 |
| **Apolipoproteins** |  |  |  |  |  |  |  |  |  |  |  |  |  |  |  |  |  |  |  |  |
| ApoA1 (g/L) | 1.18 | ± | 0.26 | 1.20 | ± | 0.27 | 0.02 | ± | 0.01 | 1.17 | ± | 0.26 | 1.14 | ± | 0.26 | -0.03 | ± | 0.02 | 0.08 | 0.04 |
| ApoB (g/L) | 0.76 | ± | 0.17 | 0.81 | ± | 0.18 | 0.04 | ± | 0.02 | 0.74 | ± | 0.17 | 0.73 | ± | 0.16 | -0.02 | ± | 0.02 | 0.01 | 0.02 |
| ApoB/ApoA1 (x10^^2^) | 65.3 | ± | 14.6 | 67.6 | ± | 15.2 | 2.35 | ± | 1.20 | 64.4 | ± | 14.4 | 64.1 | ± | 14.3 | -0.29 | ± | 1.04 | 0.05 | 0.21 |
| **Fatty acids** |  |  |  |  |  |  |  |  |  |  |  |  |  |  |  |  |  |  |  |  |
| TotFA | 9.04 | ± | 2.01 | 9.47 | ± | 2.10 | 0.44 | ± | 0.25 | 8.65 | ± | 1.94 | 8.50 | ± | 1.90 | -0.16 | ± | 0.20 | 0.07 | 0.07 |
| UnSat (x10^^2^) | 110 | ± | 24.6 | 112 | ± | 25.0 | 1.73 | ± | 0.84 | 110 | ± | 24.7 | 112 | ± | 25.0 | 1.22 | ± | 0.91 | 0.64 | 0.74 |
| DHA (x10^^2^) | 8.70 | ± | 1.93 | 10.0 | ± | 2.21 | 1.75 | ± | 0.85 | 9.07 | ± | 2.03 | 8.77 | ± | 1.96 | -0.30 | ± | 0.46 | 0.04 | 0.09 |
| LA | 2.50 | ± | 0.55 | 2.65 | ± | 0.59 | 0.15 | ± | 0.06 | 2.39 | ± | 0.54 | 2.38 | ± | 0.53 | -0.02 | ± | 0.08 | 0.07 | 0.10 |
| FAw3 | 0.28 | ± | 0.06 | 0.31 | ± | 0.07 | 0.04 | ± | 0.01 | 0.27 | ± | 0.06 | 0.27 | ± | 0.06 | 0.002 | ± | 0.02 | 0.04 | 0.11 |
| FAw6 | 3.02 | ± | 0.67 | 3.18 | ± | 0.71 | 0.16 | ± | 0.07 | 2.92 | ± | 0.65 | 2.87 | ± | 0.64 | -0.05 | ± | 0.08 | 0.03 | 0.04 |
| PUFA | 3.30 | ± | 0.73 | 3.50 | ± | 0.78 | 0.20 | ± | 0.08 | 3.19 | ± | 0.71 | 3.14 | ± | 0.70 | -0.05 | ± | 0.10 | 0.03 | 0.05 |
| MUFA | 2.27 | ± | 0.51 | 2.36 | ± | 0.52 | 0.09 | ± | 0.08 | 2.21 | ± | 0.49 | 2.17 | ± | 0.49 | -0.04 | ± | 0.06 | 0.23 | 0.15 |
| SFA | 3.41 | ± | 0.76 | 3.53 | ± | 0.78 | 0.12 | ± | 0.10 | 3.25 | ± | 0.73 | 3.18 | ± | 0.71 | -0.07 | ± | 0.06 | 0.12 | 0.10 |
| DHA/FA (%) | 1.00 | ± | 0.05 | 1.05 | ± | 0.06 | 0.04 | ± | 0.04 | 1.04 | ± | 0.05 | 1.02 | ± | 0.05 | -0.02 | ± | 0.04 | 0.15 | 0.15 |
| LA/FA (%) | 27.7 | ± | 0.40 | 28.0 | ± | 0.42 | 0.36 | ± | 0.33 | 27.6 | ± | 0.41 | 28.0 | ± | 0.33 | 0.37 | ± | 0.36 | 0.99 | 0.68 |
| FAw3/FA (%) | 3.04 | ± | 0.13 | 3.27 | ± | 0.17 | 0.22 | ± | 0.11 | 3.06 | ± | 0.14 | 3.14 | ± | 0.14 | 0.08 | ± | 0.12 | 0.26 | 0.67 |
| FAw6/FA (%) | 33.5 | ± | 0.39 | 33.7 | ± | 0.33 | -3.14 | ± | 2.42 | 33.7 | ± | 0.37 | 33.8 | ± | 0.33 | 0.12 | ± | 0.32 | 0.19 | 0.17 |
| PUFA/FA (%) | 36.5 | ± | 0.44 | 37.0 | ± | 0.56 | 0.44 | ± | 0.46 | 36.8 | ± | 0.47 | 37.0 | ± | 0.39 | 0.20 | ± | 0.41 | 0.67 | 0.93 |
| MUFA/FA (%) | 25.7 | ± | 0.40 | 25.7 | ± | 0.47 | 0.001 | ± | 0.28 | 25.5 | ± | 0.36 | 25.5 | ± | 0.35 | -0.03 | ± | 0.30 | 0.94 | 0.84 |
| SFA/FA (%) | 37.8 | ± | 0.33 | 37.4 | ± | 0.32 | -0.44 | ± | 0.28 | 37.7 | ± | 0.30 | 37.5 | ± | 0.22 | -0.18 | ± | 0.25 | 0.47 | 0.72 |
| **Glycolysis related metabolites** | | | |  |  |  |  |  |  |  |  |  |  |  |  |  |  |  |  |  |
| Glc | 4.29 | ± | 0.15 | 4.02 | ± | 0.11 | -0.47 | ± | 0.28 | 4.06 | ± | 0.17 | 3.59 | ± | 0.08 | -0.27 | ± | 0.25 | 0.56 | 0.21 |
| Lac | 1.19 | ± | 0.06 | 1.26 | ± | 0.07 | 0.07 | ± | 0.06 | 1.30 | ± | 0.08 | 1.16 | ± | 0.06 | -0.07 | ± | 0.09 | 0.05 | 0.19 |
| Pyr (x10^^-2^) | 4.70 | ± | 0.39 | 3.95 | ± | 0.27 | -0.10 | ± | 0.48 | 4.74 | ± | 0.38 | 4.05 | ± | 0.43 | -0.17 | ± | 0.49 | 0.92 | 0.66 |
| Cit (x10^^-2^) | 9.29 | ± | 0.38 | 9.54 | ± | 0.43 | 0.25 | ± | 0.27 | 9.61 | ± | 0.40 | 10.1 | ± | 0.40 | 1.92 | ± | 0.95 | 0.12 | 0.03 |
| Glol (x10^^-2^) | 10.3 | ± | 1.19 | 6.97 | ± | 0.49 | -4.39 | ± | 1.61 | 8.77 | ± | 0.54 | 6.27 | ± | 0.34 | -2.24 | ± | 0.64 | 0.23 | 0.05 |
| **Amino acids** |  |  |  |  |  |  |  |  |  |  |  |  |  |  |  |  |  |  |  |  |
| Ala (x10^^-2^) | 39.5 | ± | 1.74 | 37.8 | ± | 1.72 | -3.56 | ± | 3.11 | 38.6 | ± | 1.61 | 36.5 | ± | 1.87 | -0.19 | ± | 3.44 | 0.60 | 0.59 |
| Gln (x10^^-2^) | 31.8 | ± | 1.38 | 34.0 | ± | 1.45 | 1.21 | ± | 0.62 | 30.5 | ± | 1.54 | 33.8 | ± | 1.47 | 5.18 | ± | 2.80 | 0.20 | 0.70 |
| Gly (x10^^-2^) | 24.9 | ± | 0.96 | 25.2 | ± | 0.85 | 0.23 | ± | 1.91 | 26.2 | ± | 0.99 | 26.1 | ± | 1.12 | -0.10 | ± | 3.10 | 0.94 | 0.79 |
| His (x10^^-2^) | 7.07 | ± | 0.18 | 7.21 | ± | 0.26 | 0.14 | ± | 0.24 | 7.09 | ± | 0.21 | 6.84 | ± | 0.24 | 0.11 | ± | 0.52 | 0.96 | 0.97 |
| Ile (x10^^-2^) | 6.21 | ± | 0.34 | 6.48 | ± | 0.34 | -0.05 | ± | 0.58 | 6.32 | ± | 0.38 | 6.51 | ± | 0.26 | -0.14 | ± | 0.64 | 0.88 | 0.07 |
| Leu (x10^^-2^) | 8.32 | ± | 0.38 | 9.05 | ± | 0.45 | 0.62 | ± | 0.36 | 8.30 | ± | 0.40 | 8.55 | ± | 0.32 | 0.64 | ± | 0.58 | 0.98 | 0.61 |
| Val (x10^^-2^) | 17.3 | ± | 0.95 | 19.0 | ± | 0.81 | 0.78 | ± | 1.66 | 17.3 | ± | 0.97 | 17.9 | ± | 0.92 | 1.51 | ± | 1.80 | 0.83 | 0.91 |
| Phe (x10^^-2^) | 7.62 | ± | 0.29 | 7.64 | ± | 0.31 | 0.02 | ± | 0.23 | 7.69 | ± | 0.28 | 7.46 | ± | 0.36 | 0.55 | ± | 0.81 | 0.56 | 0.37 |
| Tyrosine (x10^^-2^) | 6.15 | ± | 1.38 | 6.46 | ± | 1.44 | 0.31 | ± | 0.26 | 6.48 | ± | 1.45 | 5.77 | ± | 1.29 | -0.71 | ± | 0.25 | 0.03 | 0.01 |
| Ace (x10^^-2^) | 7.96 | ± | 0.49 | 7.71 | ± | 0.58 | -0.25 | ± | 0.58 | 8.05 | ± | 0.56 | 6.30 | ± | 0.40 | -1.74 | ± | 0.67 | 0.12 | 0.03 |
| AcAce (x10^^-2^) | 3.21 | ± | 0.33 | 3.33 | ± | 0.42 | 0.28 | ± | 0.48 | 3.35 | ± | 0.45 | 3.31 | ± | 0.41 | -0.04 | ± | 0.56 | 0.66 | 0.44 |
| bOHBut (x10^^-2^) | 14.0 | ± | 0.92 | 13.5 | ± | 0.97 | -1.09 | ± | 1.37 | 14.6 | ± | 0.82 | 14.8 | ± | 1.01 | 0.22 | ± | 1.64 | 0.57 | 0.17 |
| Crea (x10^^-2^) | 6.66 | ± | 0.29 | 6.48 | ± | 0.24 | -0.17 | ± | 0.18 | 6.68 | ± | 0.29 | 6.66 | ± | 0.20 | -0.02 | ± | 0.23 | 0.64 | 0.89 |
| Alb (x10^^-2^) | 8.37 | ± | 0.11 | 8.40 | ± | 0.12 | 0.03 | ± | 0.10 | 8.49 | ± | 0.13 | 8.44 | ± | 0.09 | 0.38 | ± | 0.43 | 0.46 | 0.72 |
| Gp (x10^^-2^) | 1.17 | ± | 0.02 | 1.22 | ± | 0.03 | 0.05 | ± | 0.02 | 1.21 | ± | 0.03 | 1.19 | ± | 0.03 | -0.02 | ± | 0.03 | 0.04 | 0.01 |
| **Fatty acids** |  |  |  |  |  |  |  |  |  |  |  |  |  |  |  |  |  |  |  |  |
| C14:0 | 5.11 | ± | 0.37 | 4.81 | ± | 0.34 | -0.29 | ± | 0.41 | 5.01 | ± | 0.34 | 5.29 | ± | 0.26 | 0.28 | ± | 0.37 | 0.27 | 0.09 |
| C16:0 | 26.2 | ± | 0.5 | 26.0 | ± | 0.56 | -0.22 | ± | 0.37 | 26.0 | ± | 0.42 | 26.1 | ± | 0.50 | 0.10 | ± | 0.38 | 0.59 | 0.79 |
| C16:1n7cis | 3.69 | ± | 0.17 | 3.61 | ± | 0.16 | -0.08 | ± | 0.22 | 3.60 | ± | 0.15 | 3.85 | ± | 0.16 | 0.24 | ± | 0.17 | 0.25 | 0.03 |
| C18:0 | 12.8 | ± | 0.33 | 12.7 | ± | 0.36 | -0.12 | ± | 0.15 | 12.5 | ± | 0.34 | 12.2 | ± | 0.38 | -0.28 | ± | 0.36 | 0.67 | 0.59 |
| C18:1n9cis | 14.8 | ± | 0.57 | 14.6 | ± | 0.53 | -0.20 | ± | 0.30 | 15.3 | ± | 0.48 | 15.1 | ± | 0.45 | -0.17 | ± | 0.49 | 0.96 | 0.85 |
| C18:2n6 | 22.4 | ± | 0.53 | 23.1 | ± | 0.55 | 0.72 | ± | 0.43 | 23.2 | ± | 0.69 | 22.5 | ± | 0.55 | -0.69 | ± | 0.56 | 0.11 | 0.13 |
| C18:3n6 | 0.30 | ± | 0.02 | 0.30 | ± | 0.02 | 0.001 | ± | 0.02 | 0.32 | ± | 0.02 | 0.27 | ± | 0.02 | -0.06 | ± | 0.02 | 0.06 | 0.14 |
| C18:3n3 | 0.47 | ± | 0.03 | 0.51 | ± | 0.03 | 0.04 | ± | 0.04 | 0.48 | ± | 0.03 | 0.50 | ± | 0.02 | 0.02 | ± | 0.03 | 0.42 | 0.70 |
| C20:0 | 0.80 | ± | 0.06 | 0.77 | ± | 0.06 | -0.03 | ± | 0.06 | 0.78 | ± | 0.06 | 0.85 | ± | 0.06 | 0.08 | ± | 0.06 | 0.22 | 0.08 |
| C20:1n9 | 0.18 | ± | 0.01 | 0.17 | ± | 0.01 | -0.02 | ± | 0.01 | 0.17 | ± | 0.01 | 0.18 | ± | 0.01 | 0.01 | ± | 0.01 | 0.12 | 0.06 |
| C20:2n6 | 0.21 | ± | 0.01 | 0.23 | ± | 0.01 | 0.01 | ± | 0.01 | 0.23 | ± | 0.01 | 0.20 | ± | 0.01 | -0.02 | ± | 0.01 | 0.39 | 0.35 |
| C20:3n6 | 1.86 | ± | 0.11 | 1.80 | ± | 0.08 | -0.05 | ± | 0.06 | 1.74 | ± | 0.09 | 1.55 | ± | 0.10 | -0.19 | ± | 0.08 | 0.23 | 0.17 |
| C20:4n6 | 7.08 | ± | 0.34 | 7.33 | ± | 0.36 | 0.25 | ± | 0.27 | 6.85 | ± | 0.32 | 7.06 | ± | 0.31 | 0.21 | ± | 0.18 | 0.88 | 0.55 |
| C20:5n3 | 0.58 | ± | 0.04 | 0.57 | ± | 0.04 | -0.01 | ± | 0.02 | 0.54 | ± | 0.04 | 0.52 | ± | 0.04 | -0.02 | ± | 0.03 | 0.67 | 0.76 |
| C22:0 | 0.15 | ± | 0.01 | 0.14 | ± | 0.01 | -0.01 | ± | 0.01 | 0.14 | ± | 0.01 | 0.17 | ± | 0.01 | 0.02 | ± | 0.01 | 0.02 | 0.03 |
| C24:0 | 0.37 | ± | 0.05 | 0.32 | ± | 0.04 | -0.06 | ± | 0.06 | 0.30 | ± | 0.04 | 0.38 | ± | 0.05 | 0.08 | ± | 0.05 | 0.10 | 0.03 |
| C22:6n3 | 1.24 | ± | 0.08 | 1.34 | ± | 0.09 | 0.10 | ± | 0.07 | 1.17 | ± | 0.09 | 1.41 | ± | 0.12 | 0.24 | ± | 0.06 | 0.13 | 0.88 |
| C24:1n9 | 0.12 | ± | 0.01 | 0.11 | ± | 0.01 | -0.01 | ± | 0.01 | 0.12 | ± | 0.01 | 0.14 | ± | 0.01 | 0.02 | ± | 0.01 | 0.08 | 0.02 |

All concentrations are mmol/L. P^1^ – Delta statistics unadjusted. P^2^ – Delta statistics adjusted for weight change. Values are reported as mean ± SE. On treatment analysis was performed for all variables. The level of significance was set to p-value <0.05 after Bonferroni correction. Baseline samples collected at visit 1, post treatment samples were collected at visit 6 (5 weeks of treatment total). XXL-VLDL-P, Concentration of chylomicrons and extremely large VLDL particles; XXL-VLDL-L, Total lipids in chylomicrons and extremely large VLDL; XXL-VLDL-C, Total cholesterol in chylomicrons and extremely large VLDL; XXL-VLDL-CE, Cholesterol esters in chylomicrons and extremely large VLDL; XXL-VLDL-FC, Free cholesterol in chylomicrons and extremely large VLDL; XXL-VLDL-TG, Triglycerides in chylomicrons and extremely large VLDL; XL-VLDL-P, Concentration of very large VLDL particles; XL-VLDL-L, Total lipids in very large VLDL; XL-VLDL-PL, Phospholipids in very large VLDL; XL-VLDL-C, Total cholesterol in very large VLDL; XL-VLDL-CE, Cholesterol esters in very large VLDL; XL-VLDL-FC, Free cholesterol in very large VLDL; XL-VLDL-TG, Triglycerides in very large VLDL; L-VLDL-P, Concentration of large VLDL particles; L-VLDL-L, Total lipids in large VLDL; L-VLDL-PL Phospholipids in large VLDL; L-VLDL-C, Total cholesterol in large VLDL; L-VLDL-CE, Cholesterol esters in large VLDL; L-VLDL-FC, Free cholesterol in large VLDL; L-VLDL-TG, Triglycerides in large VLDL; M-VLDL-P, Concentration of medium VLDL particles; M-VLDL-L, Total lipids in medium VLDL; M-VLDL-PL, Phospholipids in medium VLDL; M-VLDL-C, Total cholesterol in medium M-VLDL-CE, Cholesterol esters in medium VLDL; M-VLDL-FC, Free cholesterol in medium VLDL; M-VLDL-TG, Triglycerides in medium VLDL; S-VLDL-P, Concentration of small VLDL particles; S-VLDL-L, Total lipids in small VLDL; S-VLDL-PL, Phospholipids in small VLDL; S-VLDL-C, Total cholesterol in small VLDL; S-VLDL-CE, Cholesterol esters in small VLDL; S-VLDL-FC, Free cholesterol in small VLDL; S-VLDL-TG, Triglycerides in small VLDL; XS-VLDL-P, Concentration of very small VLDL particles; XS-VLDL-L, Total lipids in very small VLDL; XS-VLDL-PL, Phospholipids in very small VLDL; XS-VLDL-C, Total cholesterol in very small VLDL; XS-VLDL-CE, Cholesterol esters in very small VLDL; XS-VLDL-FC, Free cholesterol in very small VLDL; XS-VLDL-TG, Triglycerides in very small VLDL; IDL-P, Concentration of IDL particles IDL-L, Total lipids in IDL; IDL-PL, Phospholipids in IDL; IDL-C, Total cholesterol in IDL; IDL-CE, Cholesterol esters in IDL; IDL-FC, Free cholesterol in IDL; IDL-TG, Triglycerides in IDL; L-LDL-P, Concentration of large LDL particles; L-LDL-L, Total lipids in large LDL; L-LDL-PL, Phospholipids in large LDL; L-LDL-C, Total cholesterol in large LDL; L-LDL-CE, Cholesterol esters in large LDL; L-LDL-FC, Free cholesterol in large LDL; L-LDL-TG, Triglycerides in large LDL; M-LDL-P, Concentration of medium LDL particles; M-LDL-L, Total lipids in medium LDL; M-LDL-PL, Phospholipids in medium LDL; M-LDL-C, Total cholesterol in medium LDL; M-LDL-CE, Cholesterol esters in medium LDL; M-LDL-FC, Free cholesterol in medium LDL; M-LDL-TG, Triglycerides in medium LDL; S-LDL-P, Concentration of small LDL particles; S-LDL-L, Total lipids in small LDL; S-LDL-PL, Phospholipids in small LDL; S-LDL-C, Total cholesterol in small LDL; S-LDL-CE, Cholesterol esters in small LDL; S-LDL-FC, Free cholesterol in small LDL; S-LDL-TG, Triglycerides in small LDL; XL-HDL-P, Concentration of very large HDL particles; XL-HDL-L, Total lipids in very large HDL; XL-HDL-PL, Phospholipids in very large HDL; XL-HDL-C, Total cholesterol in very large HDL; XL-HDL-CE, Cholesterol esters in very large HDL; XL-HDL-FC, Free cholesterol in very large HDL; XL-HDL-TG, Triglycerides in very large HDL; L-HDL-P, Concentration of large HDL particles; L-HDL-L, Total lipids in large HDL; L-HDL-PL, Phospholipids in large HDL; L-HDL-C, Total cholesterol in large HDL; L-HDL-CE, Cholesterol esters in large HDL; L-HDL-FC, Free cholesterol in large HDL; L-HDL-TG, Triglycerides in large HDL; M-HDL-P, Concentration of medium HDL particles; M-HDL-L, Total lipids in medium HDL; M-HDL-PL, Phospholipids in medium HDL; M-HDL-C, Total cholesterol in medium HDL; M-HDL-CE, Cholesterol esters in medium HDL; M-HDL-FC, Free cholesterol in medium HDL; M-HDL-TG, Triglycerides in medium HDL; S-HDL-P, Concentration of small HDL particles; S-HDL-L, Total lipids in small HDL; S-HDL-PL, Phospholipids in small HDL; S-HDL-C, Total cholesterol in small HDL; S-HDL-CE, Cholesterol esters in small HDL; S-HDL-FC, Free cholesterol in small HDL; S-HDL-TG, Phospholipids to total lipds ratio in chylomicrons and extremely large VLDL; XXL-VLDL-C_%, Total cholesterol to total lipids ratio in chylomicrons and extremely large VLDL; XXL-VLDL-CE_%, Cholesterol esters to total lipids ratio in chylomicrons and extremely large VLDL; XXL-VLDL-FC_%, Free cholesterol to total lipids ratio in chylomicrons and extremely large VLDL; XXL-VLDL-TG_%, Triglycerides to total lipids ratio in chylomicrons and extremely large VLDL; XL-VLDL-PL_%, Phospholipids to total lipds ratio in very large VLDL; XL-VLDL-C_%, Total cholesterol to total lipids ratio in very large VLDL; XL-VLDL-CE_%, Cholesterol esters to total lipids ratio in very large VLDL; XL-VLDL-FC_%, Free cholesterol to total lipids ratio in very large VLDL; XL-VLDL-TG_%, Triglycerides to total lipids ratio in very large VLDL; L-VLDL-PL_%, Phospholipids to total lipids ratio in large VLDL; L-VLDL-C_%, Total cholesterol to total lipids ratio in large VLDL; L-VLDL-CE_%, Cholesterol esters to total lipids ratio in large VLDL; L-VLDL-FC_%, Free cholesterol to total lipids ratio in large VLDL; L-VLDL-TG_%, Triglycerides to total lipids ratio in large VLDL; M-VLDL-PL_%, Phospholipids to total lipids ratio in medium VLDL; M-VLDL-C_%, Total cholesterol to total lipids ratio in medium VLDL; M-VLDL-CE_%, Cholesterol esters to total lipids ratio in medium VLDL; M-VLDL-FC_%, Free cholesterol to total lipids ratio in medium VLDL; M-VLDL-TG_%, Triglycerides to total lipids ratio in medium VLDL; S-VLDL-PL_%, Phospholipids to total lipids ratio in small VLDL; S-VLDL-C_%, Total cholesterol to total lipids ratio in small VLDL; S-VLDL-CE_%, Cholesterol esters to total lipids ratio in small VLDL; S-VLDL-FC_%, Free cholesterol to total lipids ratio in small VLDL; S-VLDL-TG_%, Triglycerides to total lipids ratio in small VLDL; XS-VLDL-PL_%, Phospholipids to total lipids ratio in very small VLDL; XS-VLDL-C_%, Total cholesterol to total lipids ratio in very small VLDL; XS-VLDL-CE_%, Cholesterol esters to total lipids ratio in very small VLDL; XS-VLDL-FC_%, Free cholesterol to total lipids ratio in very small VLDL; XS-VLDL-TG_%, Triglycerides to total lipids ratio in very small VLDL; IDL-PL_%, Phospholipids to total lipids ratio in IDL; IDL-C_%, Total cholesterol to total lipids ratio in IDL; IDL-CE_%, Cholesterol esters to total lipids ratio in IDL; IDL-FC_%, Free cholesterol to total lipids ratio in IDL; IDL-TG_%, Triglycerides to total lipids ratio in IDL; L-LDL-PL_%, Phospholipids to total lipids ratio in large LDL; L-LDL-C_%, Total cholesterol to total lipids ratio in large LDL; L-LDL-CE_%, Cholesterol esters to total lipids ratio in large LDL; L-LDL-FC_%, Free cholesterol to total lipids ratio in large LDL; L-LDL-TG_%, Triglycerides to total lipids ratio in large LDL; M-LDL-PL_%, Phospholipids to total lipids ratio in medium LDL; M-LDL-C_%, Total cholesterol to total lipids ratio in medium LDL; M-LDL-CE_%, Cholesterol esters to total lipids ratio in medium LDL; M-LDL-FC_%, Free cholesterol to total lipids ratio in medium LDL; M-LDL-TG_%, Triglycerides to total lipids ratio in medium LDL; S-LDL-PL_%, Phospholipids to total lipids ratio in small LDL; S-LDL-C_%, Total cholesterol to total lipids ratio in small LDL; S-LDL-CE_%, Cholesterol esters to total lipids ratio in small LDL; S-LDL-FC_%, Free cholesterol to total lipids ratio in small LDL; S-LDL-TG_%, Triglycerides to total lipids ratio in small LDL; XL-HDL-PL_%, Phospholipids to total lipids ratio in very large HDL; XL-HDL-C_%, Total cholesterol to total lipids ratio in very large HDL; XL-HDL-CE_%, Cholesterol esters to total lipids ratio in very large HDL; XL-HDL-FC_%, Free cholesterol to total lipids ratio in very large HDL; XL-HDL-TG_%, Triglycerides to total lipids ratio in very large HDL; L-HDL-PL_%, Phospholipids to total lipids ratio in large HDL; L-HDL-C_%, Total cholesterol to total lipids ratio in large HDL; L-HDL-CE_%, Cholesterol esters to total lipids ratio in large HDL; L-HDL-FC_%, Free cholesterol to total lipids ratio in large HDL; L-HDL-TG_%, Triglycerides to total lipids ratio in large HDL; M-HDL-PL_%, Phospholipids to total lipids ratio in medium HDL; M-HDL-C_%, Total cholesterol to total lipids ratio in medium HDL; M-HDL-CE_%, Cholesterol esters to total lipids ratio in medium HDL; M-HDL-FC_%, Free cholesterol to total lipids ratio in medium HDL; M-HDL-TG_%, Triglycerides to total lipids ratio in medium HDL; S-HDL-PL_%, Phospholipids to total lipids ratio in small HDL; S-HDL-C_%, Total cholesterol to total lipids ratio in small HDL; S-HDL-CE_%, Cholesterol esters to total lipids ratio in small HDL; S-HDL-FC_%, Free cholesterol to total lipids ratio in small HDL; S-HDL-TG_%, Triglycerides to total lipids ratio in small HDL; VLDL-D, Mean diameter for VLDL particles; LDL-D, Mean diameter for LDL particles; HDL-D, Mean diameter for HDL particles; Serum-C, Serum total cholesterol; VLDL-C, Total cholesterol in VLDL; Remnant-C, Remnant cholesterol (non-HDL, non-LDL -cholesterol); LDL-C, Total cholesterol in LDL; HDL-C, Total cholesterol in HDL; HDL2-C, Total cholesterol in HDL2; HDL3-C, Total cholesterol in HDL3; EstC, Esterified cholesterol; FreeC, Free cholesterol; Serum-TG, Serum total triglycerides; VLDL-TG, Triglycerides in VLDL; LDL-TG, Triglycerides in LDL; HDL-TG, Triglycerides in HDL; TotPG, Total phosphoglycerides; TG/PG, Ratio of triglycerides to phosphoglycerides; PC, Phosphatidylcholine and other cholines; SM, Sphingomyelins; TotCho, Total cholines; ApoA1, Apolipoprotein A-I; ApoB, Apolipoprotein B; ApoB/ApoA1, Ratio of apolipoprotein B to apolipoprotein A-I; TotFA, Total fatty acids; UnSat, Estimated degree of unsaturation; DHA, 22:6, docosahexaenoic acid; LA, 18:2, linoleic acid; FAw3, Omega-3 fatty acids; FAw6, Omega-6 fatty acids; PUFA, Polyunsaturated fatty acids; MUFA, Monounsaturated fatty acids; 16:1, 18:1; SFA, Saturated fatty acids; DHA/FA, Ratio of 22:6 docosahexaenoic acid to total fatty acids; LA/FA, Ratio of 18:2 linoleic acid to total fatty acids; FAw3/FA, Ratio of omega-3 fatty acids to total fatty acids; Aw6/FA, Ratio of omega-6 fatty acids to total fatty acids; PUFA/FA, Ratio of polyunsaturated fatty acids to total fatty acids; MUFA/FA, Ratio of monounsaturated fatty acids to total fatty acids; SFA/FA, Ratio of saturated fatty acids to total fatty acids; Glc, Glucose; Lac, Lactate;Pyr, Py ruvate;Cit, Ci trate; Glol, Glycerol; Ala, Alanine; Gln, Glutamine; Gly, Glycine; His, Histidine; Ile, Isoleucine; Leu, Leucine; Val, Valine; Phe, Phenylalanine; Tyr, Tyrosine; Ace, Acetate; AcAce, Acetoacetate; bOHBut, 3-hydroxybutyrate; Crea, Creatinine; Alb, Albumin; Gp, Glycoprotein acetyls, mainly a1-acid.
